# Systemic and gut microbiome changes with metformin and liraglutide in youth-onset type 2 diabetes: the MIGHTY study

**DOI:** 10.1080/19490976.2025.2558071

**Published:** 2025-09-29

**Authors:** Sophia B. Glaros, Sidharth P. Mishra, Shalini Jain, Faith S. Davis, Scott A. Gabel, Geoffrey A. Mueller, Alan K. Jarmusch, Lilian Mabundo, Amber B. Courville, Mary F. Walter, Peter J. Walter, Kirsten E. Overdahl, Hariom Yadav, Stephanie T. Chung

**Affiliations:** a Diabetes, Endocrinology, and Obesity Branch, National Institute of Diabetes and Digestive and Kidney Diseases, National Institutes of Health, Bethesda, MD, USA; b USF Center for Microbiome Research, Microbiomes Institute, University of South Florida Morsani College of Medicine, Tampa, FL, USA; c Department of Neurosurgery and Brain Repair, University of South Florida Morsani College of Medicine, Tampa, FL, USA; d Nuclear Magnetic Resonance Group, National Institute of Environmental Health Sciences, Durham, NC, USA; e Metabolomics Core Facility, Inflammation, Immunity and Disease Laboratory, National Institute of Environmental Health Sciences, Durham, NC, USA; f Clinical Laboratory Services Core, National Institute of Diabetes and Digestive and Kidney Diseases, National Institutes of Health, Bethesda, MD, USA; g Clinical Mass Spectrometry Core, National Institute of Diabetes and Digestive and Kidney Diseases, National Institutes of Health, Bethesda, MD, USA; h Division of Diabetes and Endocrinology, Children’s National Hospital, Washington, DC, USA

**Keywords:** Diabetes, microbiome, metformin, youth-onset type 2 diabetes, glucagon-like peptide-1 receptor agonist, short-chain fatty acid, bile acid, metabolomics

## Abstract

Metformin (Met) and liraglutide (Lira) are preferred diabetes therapies that may improve glycemia by modulating the gut microbiome, but the mechanisms and pathways are unknown and few data exist in youth-onset type 2 diabetes (Y-T2D). In a 3-month parallel clinical trial in African American Y-T2D randomized to Met (*n* = 14) or Met+Lira (*n* = 11), we compared gut microbial composition and metabolomic profiles and determined the relationship of changes in microbial abundance with glycemia and plasma metabolites. After 3 months, Met was associated with greater relative abundance of *Eubacterium* and *Eubacterium rectale* and lower *Bacteroides ovates* (*p* < 0.05). Met+Lira was associated with greater *Bacteroides fragilis* and lower *Streptococcus thermophilus* (*p* < 0.05). Met group had increased (>1.5-fold) plasma cholic secondary bile acids (sulfochenodeoxycholic acid, nutriacholic acid, alpha-muricholic acid, and C24 dihydroxy bile acid; *p* ≤ 0.002). The change in nutriacholic acid correlated with lower fasting glucose (*r* = −0.7, *p* < 0.05). Shifts in microbiota taxa were not associated with plasma short-chain fatty acids (SCFA), hemoglobin A1c or glucose. Short-term Met and Met+Lira in Y-T2D were related to distinct shifts toward bile acid and SCFA-producing gut microbiota taxa, and secondary bile acid metabolites correlated with improved glycemia, suggesting bile acid pathways may be important modulators of glycemia in youth on metformin.

**Clinical trials.gov identifier**: NCT02960659

## GRAPHICAL ABSTRACT

**Figure:**
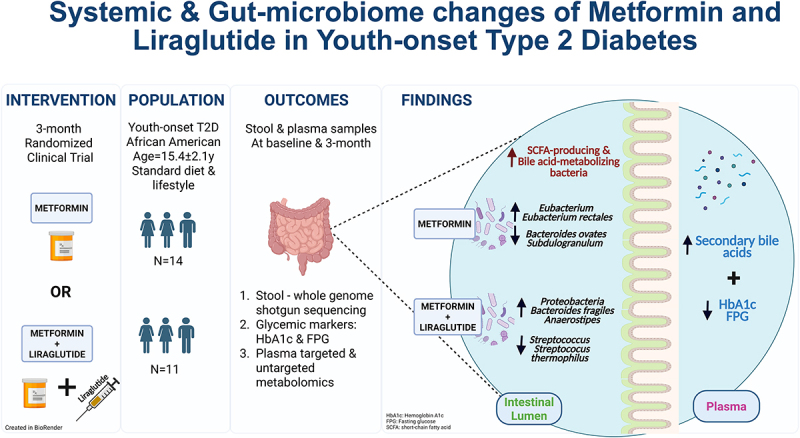

## Introduction

Gut dysbiosis – characterized by reduced microbial diversity and decreased abundance of short-chain fatty-acid (SCFA) producing bacteria – is a distinctive feature of type 2 diabetes in adults and may be intricately linked to severe hyperglycemia and poor response to first-line treatment in youth-onset type 2 diabetes (Y-T2D).^1–3^ In Y-T2D, metformin (Met) is the recommended first-line therapy, but up to 50% of youth experience monotherapy failure necessitating treatment with alternate agents, such as liraglutide (Lira), a glucagon-like peptide −1 receptor agonist GLP-1 RA.^4,5^ Treatment responsiveness could depend on targeted shifts in gut microbial abundance and metabolism, but data are sparse in youth.^3,6,7^ In adults, metformin and liraglutide are associated with restoration of gut microbial homeostasis^8,9^; however, elucidating medication-related changes in the gut microbiome has been challenging because of the heterogeneity of type 2 diabetes and the diversity of the human microbiota.^2,10^

Emerging data supports the primary role of the gut microbiome in mediating disease responsiveness and drug metabolism.^11,12^ However, inconsistent drug-induced shifts toward a healthier gut microbial profile are influenced by myriad upstream socio-environmental factors that moderate the gut microbiota. While most studies have been conducted in adults, these findings may not be applicable to Y-T2D, as unique contextual factors in adolescents may influence microbial composition and function. Youth have a more aggressive T2D pathogenesis with rapid deterioration in glycemic control and unique age-related environmental and cultural habits.^13^ Dynamic age-related changes in the composition and diversity of the microbiota across the lifecourse alter the balance of intestinal microbes and host energy metabolism in adolescents compared to adults.^14,15^ In addition to metabolic derangements observed in Y-T2D,^16^ the unique gut microbial architecture in Y-T2D may be impacted further by combination therapy with liraglutide,^17^ host factors (e.g., puberty, diet, exercise),^18^ and environmental factors (culture, country, geographic location).^15,19^ Furthermore, youth with obesity have been observed to have lower fiber intake and a reduced abundance of short-chain fatty acid (SCFA) producing bacteria which may be critical mediators in metformin treatment.^18,20^

Understanding these mechanistic pathways is important for establishing causality. Metformin and liraglutide may facilitate systemic glucose homeostasis by upregulating SCFA^8^ and bile-acid metabolism, but the exact pathways linking microbial shifts with metabolic pathways remain unclear.^21,22^ In adults, metformin inconsistently increased the relative abundance of SCFA-producing and bile-acid metabolizing bacteria in some,^23–27^ but not all studies,^28,29^ with a variable relationship to improved glycemia. Metformin-induced changes in youth are even less well understood, with only two studies yielding conflicting results.^6,30^ In one study, no consistent shifts in overall gut microbial architecture were observed among youth with obesity on metformin.^30^ While in a small pilot study from our lab, metformin was associated with a shift toward SCFA-producing organisms (*Akkermansia* and *Enterobacteriaceae*) in Y-T2D, though the effect size was small and changes were not related to glycemia or metabolites.^6^ Fewer analyses have examined medication interactions with liraglutide and none have been conducted in youth. Liraglutide enriched intestinal bacterial communities and promoted SCFA-producing species in mice^31^ and adult humans,^9,32^ though the mechanisms for these changes remain elusive.

This study was designed to investigate entero-systemic pathways of metformin and liraglutide in a well-characterized cohort of African American Y-T2D to provide novel evidence of short-term medication-induced systemic and gut microbiome changes in a free-living environment. By conducting deep phenotyping multi-omics studies in youth with similar cultural and geographic backgrounds, we aimed to uncover potential mechanisms and treatment pathways among Y-T2D, who are understudied in this field but have high disease burden and treatment failure.^33,34^ Our goals were to characterize the relationship between intestinal microbial microarchitecture and function, and systemic glucose flux, incretin hormones, and metabolite measurements. The specific objectives in Y-T2D randomized to metformin only (Met) or metformin and liraglutide (Met+Lira) were to (1) compare the change in gut microbial composition and metabolomic profiles, and (2) determine the relationship of changes in microbial abundance with glucose flux rates and plasma metabolites.

## Materials and methods

### Trial design

This was a preplanned exploratory analysis of the Metformin Influences Gut Hormones in Youth (MIGHTY) study conducted at the Metabolic Clinical Research Unit at the NIH Clinical Center, Bethesda Maryland between 2017and 2022. The MIGHTY studies were designed to evaluate the pathophysiology of Y-T2D. The primary study objectives compared the change in rates of gluconeogenesis and beta-cell function and were previously reported.^35^ Supplementary S1 illustrates the participant flow diagram. Written informed consent was obtained from the parents or legal guardians, and assent from the participants prior to the start of trial-related procedures. The protocol was approved by the National Institute of Diabetes and Digestive and Kidney Diseases (NIDDK) Institutional Review Boards and registered at ClinicalTrials.gov (NCT02960659).

### Participants

Eligible participants were African American (both parents and youth self-identified), age 12–25 years, diagnosed with Y-T2D by the American Diabetes Association criteria,^36^ time since diagnosis ≤5 years, hemoglobin A1c (HbA1c) ≤9% (≤75 mmol/mol), and Tanner Stage 4–5. Exclusion criteria were current or past GLP-1 agonist use, treatment with insulin within the previous 3 months, metabolic acidosis or ketonemia, positive autoantibodies to glutamic acid decarboxylase, or insulinoma-associated protein-2 (IA-2A), allergy to study medications or milk protein, hemoglobin concentration <11 g/dL, serum triglyceride concentrations ≥500 mg/dl, or body weight ≤58 or ≥205 kg.

### Trial procedures

**Figure 1.**
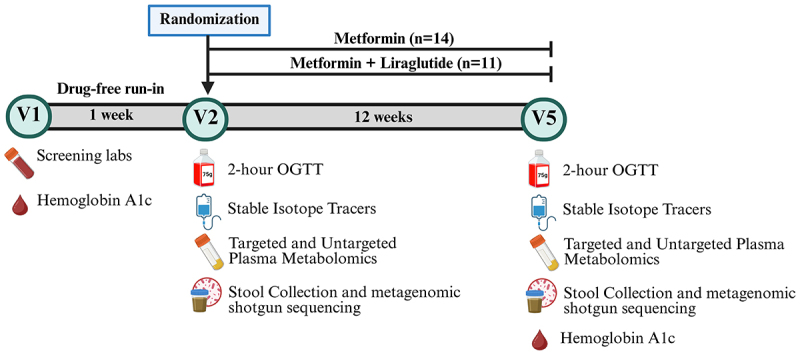
Study design: randomized parallel two-arm randomized controlled trial.

Participants were evaluated at baseline and after a three-month intervention with an identical 2-day inpatient protocol (Figure 1).^35^ Prior to the baseline visit, all glucose-lowering agents were discontinued for 5–7 days. After the baseline evaluation, participants were randomized to standard-release metformin (500 mg tablets) with or without liraglutide (0.6 mg). All participants followed a standard titration protocol to receive a maximum dose of metformin 1000 mg twice daily and liraglutide 1.8 mg daily over 3 weeks and continued for 12 weeks ±2 weeks (Supplemental materials).

Screening outpatient visit (V1) to assess eligibility. Medications (if applicable) discontinued for 5–7 days prior to baseline visit (V2), a two-day inpatient domiciled stay at the Metabolic Clinical Research Unit at the National Institutes of Health Clinical Center with diet standardization. Participants underwent a frequently sampled 75 g, 2-h oral glucose tolerance test (OGTT) with blood drawn at (0, 30, 60, 90, 120 min), stable isotope administration to measure fasting glucose production using 6,6, ^2^H_2_[glucose] and gluconeogenesis using ^2^H_2_O, plasma samples for metabolomics, and stool collection. Participants were randomized to metformin (up to 2 g daily) with or without liraglutide (up to 1.8 mg daily) for 12 weeks ±2 weeks. Participants returned for final visit (V5) and an identical two-day protocol completed. Figure created in BioRender.

On study day 1, participants were admitted to the National Institutes of Health Clinical Center after an overnight 10–12 h fast and underwent a frequently sampled 75-gram two-hour oral glucose tolerance test (OGTT) with samples at 0, 15, 30, 60, 90, and 120 min to measure plasma glucose, insulin, and C-peptide concentrations. Blood for incretin analyses were collected at 0 and 120 min, and metabolomics at 0 min. Stool samples were collected at Visits 2 (baseline) and 5 (after 12 weeks). Trough metformin concentrations were collected at 0 min (12 h post direct observed administration) at Visit 5 only. Standardized meals were administered, and participants fasted overnight (12 h) between day 1 and day 2. On study day 2, stable isotope kinetic tracers of glucose and deuterated water were administered to measure post-absorptive rates of glucose appearance and gluconeogenesis. Absolute rates of gluconeogenesis were calculated as the product of glucose appearance and fractional gluconeogenesis as previously described.^35,37^ Glucose and insulin kinetics were modeled with the Insulin Sensitivity and Secretion (ISS) model.^38^ OGTT-modeled insulin sensitivity (mS_I_, 10^−4^mU·ml·min) and β-cell function (Sigma, unitless) were estimated from the ISS (Insulin Secretion and Sensitivity model) as previously described.^38^ Due to missing glucose time points, three participants (two Met and one Met+Lira) did not have ISS modeling.

### Biochemical assays

Glucose concentrations were measured in plasma using an enzymatic hexokinase assay on the Cobas 6000 instrument (Roche Diagnostics, USA). Insulin and C-peptide were measured in serum via electrochemiluminescence on the Cobas 6000 instrument (Roche Diagnostics). HbA1c was determined using the high-performance liquid chromatography (HPLC) D10 instrument (Bio-Rad, USA). Lactate concentrations were measured using a YSI model 2900 analyzer (Yellow Springs Instrument, Yellow Springs, OH, USA). Metformin concentrations were measured with a validated liquid chromatography tandem mass spectrometry (LC–MS/MS) method (detection range 0.01–10 µg/ml). Trimethylamine-N-oxide (TMAO) was measured in EDTA plasma using a commercially available enzyme-linked immunosorbent assay (ELISA) kit from ELK Biotechnology (Denver CO, Cat. No ELK8356). The detection range was 0.16–10 μmol/L, and the Intra and Inter % coefficients of variation (CV) were 5.6 and 6.3, respectively. Venous blood for incretin analysis was collected in EDTA tubes containing protease inhibitors (Millipore-Sigma Catalog # S8830) and DPPIV inhibitors (Millipore-Sigma Catalog # DPP4–010). Plasma was separated and frozen at −80°C within 1 h of the blood draw and stored until analyses. Active glucagon like-1 peptide (GLP-1), total gastric inhibitory polypeptide (GIP), and peptide YY (PYY) were measured in plasma using the assay kits from Meso Scale Diagnostics (Rockville, MD, Catalog numbers: active GLP-1: K1503OD; Total GIP: K1515SK; PYY: K151MPD). These kits are a sandwich immunoassay that uses electrochemiluminescence Sulfo-tag^TM^ labels conjugated to detect antibodies that emit light upon electrochemical stimulation initiated at the electrode surface of the microplates. The detection range was 0.02 to 120 pM for active GLP-1, 3.7 to 12,500 pg/mL for GIP, and 2.7 to 2260 pg/mL for PYY. The intra and inter CV were 3.6% and 8.5% for active GLP-1, 3.1% and 7.1% for total GIP, and 1.3 and 2.8% for total PYY. Endogenous GLP-1 concentrations after Met+Lira intervention could not be evaluated because of the cross-reactivity of the assay with liraglutide (see Supplementary Materials).

### Stool collection and metagenomic shotgun sequencing

Stool was collected up to 24 h prior to or during Visits 2 and 5. Stool collected at home was stored in a sterile plastic vial at 4°C for <24 hours, then flash frozen with liquid nitrogen, transferred to −80°C, and stored for metagenomic stool shotgun sequencing that was conducted in single batched analysis. Briefly, genomic DNA was extracted from 150 mg of human stool samples using QIAamp PowerFecal Pro DNA Kit (Qiagen, USA) according to the manufacturer’s protocol. The extracted DNA was quantified using Qubit dsDNA HS assay kit (Thermo Fisher Scientific, USA). A total of 150 ng of the quantified DNA was used for library preparation using Illumina® DNA Prep, (M) Tagmentation kit (Illumina, Inc, 5200 Illumina Way, San Diego, CA, USA) following the manufacturer’s instructions. Sample-specific unique Illumina–Nextera DNA UD Indexes were incorporated for identification. Sequencing was carried out on Illumina NextSeq1000 platform using the Illumina NextSeq 1000/2000 P2 Reagents (300 Cycles) v3 reagent cartridge (Illumina, Inc, 5200 Illumina Way, San Diego CA, USA). All sequencing data were captured and stored in the Basespace cloud and subsequently analyzed using bioinformatics pipelines, as outlined in the bioinformatics and statistical analysis section.^39^

The analysis of shotgun sequencing data was conducted utilizing the Yet Another Metagenomic Pipeline (YAMP) workflow. The YAMP pipeline integrates tools from bbmap suite for sequence de-duplication, quality trimming, and decontamination.^40^ FastQC was employed for the visualization of both raw and QC filtered metagenomic reads (Supplemental materials). For taxonomic classification and profiling of microbial communities, the pipeline incorporates MetaPhlAn (v3.0), a marker gene-based taxonomic profiler, leverages a database of approximately 1.1 million unique clade-specific marker genes identified from ~100,000 reference genomes (~99,500 bacterial and archaeal and ~500 eukaryotic).^41^ This database enables precise taxonomic assignments, accurate estimation of organismal relative abundance, species-level resolution for bacteria, archaea, eukaryotes, and viruses. Functional profiling of the microbiome community was performed using the HUMAnN pipeline, which estimates the metabolic and functional potential of the microbial populations. Moreover, QIIME2 was utilized to compute multiple alpha diversity metrics including observed operational taxonomic units (OTUs), Shannon and Simpson diversity index.^20^ Shannon and Simpson indices were chosen to represent richness and evenness, while Chao1 was included to estimate rare taxa, given the variability in microbial complexity in youth populations. For LEfSe (Linear discriminatory analysis [LDA] Effect Size) analysis, relative abundances were calculated by normalizing amplicon sequence variants counts to the total sum of sequences per sample following rarefaction, thus accounting for differences in sequencing depth. Taxa present in fewer than a defined percentage of samples or with low relative abundance ( < 0.01%) were filtered out to reduce noise. The resulting normalized feature table was collapsed to the genus (or specified) taxonomic level and exported in a LEfSe-compatible format using the QIIME tools export command. Group metadata were formatted accordingly, and the dataset was analyzed using the LEfSe algorithm implemented via the Galaxy platform or locally, with parameters (α = 0.05 for the Kruskal–Wallis’s test and logarithmic LDA score threshold ≥3.0).

### Targeted plasma metabolomics

Frozen plasma samples were thawed on ice, and 300 µl of sample was diluted 1:1 with ^2^H-PBS with sodium azide, pH 7.4. The sample was pushed through a rinsed 3–10 kDa molecular weight cutoff (MWCO) concentrator to remove proteins. Trimethylsilypropanesulfonate (DSS) was added to a concentration of 200 µM for concentration standardization. Final sample volumes extracted were 300 µl or 500 µl and placed in Shigemi or 5 mm Wilmad NMR tubes, respectively. Excess sample was preserved for mass spectrometry (MS) analysis. NMR spectra were acquired on an 800 MHz Agilent DD2 console equipped with a cryogenically cooled probe. A 1-dimensional NOESY sequence with 4 s acquisition time, 1 s recycle delay, 256 transients, and 100 ms mixing time was used. NMR spectra were analyzed with Chenomx (Alberta, Canada).

### Untargeted metabolomics

Untargeted metabolomics was performed by the Trans-NIH Metabolomics Core. Plasma samples were prepared for analysis with a 4:1 v/v methanol crash to remove high-molecular weight proteins and other macromolecules in order to ensure a clean matrix for small-molecule analysis. Samples were analyzed using UHPLC (Vanquish, Thermo Fisher Scientific) coupled to high-resolution mass spectrometry (Orbitrap Fusion Tribrid, Thermo Fisher Scientific). Liquid Chromatography-Mass Spectrometry (LC-MS) and LC-MS/MS (liquid chromatography tandem mass spectrometry) data were acquired. LC-MS data were collected from individual samples (*n* = 1 injection), system blanks (injection of solvent used to resolubilize samples), and a pooled quality control. The pooled quality control (QC) was injected multiple times at different volumes and used in data processing. LC-MS/MS data, used to annotate features, were collected using the AcquireX (Thermo Fisher Scientific) deep scan methodology in which the pooled QC was injected multiple times (*n* = 7). Ionization was performed via heated electrospray ionization (Thermo Fisher Scientific); data were collected in both positive and negative ionization modes.

Compound Discoverer 3.3.0.550 (Thermo Scientific) was used to process.raw files in a tabular output, including descriptors of each feature (defined by m/z and retention time), annotation information (e.g. MS/MS database match), and peak area. Data were further processed using in-house R scripts via JupyterNotebooks. Data processing included formatting of the data outputs, comparison of m/z and retention time of annotation features versus an in-house generated list based on authentic chemical standards, assessment of signal response in pooled QC samples, assessment of signal variance in pooled QC samples versus samples (i.e. dispersion ratio), and multi- and univariate statistics. MSI levels of annotation confidence were provided based on the MS/MS database matching algorithm in Compound Discoverer, a list of m/z generated from authentic chemical standards, and manual annotation. The following public, commercial, and in-house MS/MS spectral libraries were used to annotate features: NIST2020, GNPS mzCloud, and in-house MS/MS spectral library acquired from authentic chemical standards purchased and run by the Metabolomics Core Facility. Features with a database or library match were putatively annotated. Four differential secondary bile acids within the cholic acids subclass were identified and reported: nutriacholic, alpha-muricholic, sulfochenodenoxycholic, and C24 dihydroxy bile acid (Supplementary Figures S2–S5). Nutriacholic was annotated via MS1 match to in-house analytical standard (Supplementary S2), alpha-muricholic acid was annotated via MS1 match to in-house analytical standard (Supplementary S3), sulfochenodeoxycholic acid was annotated via MS/MS match to public reference databases, and C24 dihydroxy bile acid was annotated via MS/MS match to in-house reference database (Supplementary S4).

### Statistical analysis

This was a pre-specified exploratory analysis. Therefore, sample size calculations were based on the study’s primary outcome that determined the change in rates of gluconeogenesis between the two treatment arms.^35^ *P*-values < 0.05 were considered statistically significant. Data are presented as mean (95% Confidence Interval) unless otherwise stated. Non-parametric data (insulin, glucagon, sigma, and mS_I_) were natural log-transformed prior to analysis. Baseline characteristics were compared between groups using Student’s t-test and Fisher's exact test. No data were imputed and only per protocol analysis performed. Glycemic outcome variables (HbA1c, fasting and two-hour glucose, and rates of gluconeogenesis) were analyzed using an ANCOVA that compared the two randomized groups adjusting for baseline levels. Correlations were assessed with Spearman correlation coefficients (r). Statistical analyses were performed with STATA, v17.0 (College Station, Texas) unless otherwise specified below.

For the microbial analysis, beta-diversity, alpha-diversity, and different taxa bacterial abundance was conducted. Beta diversity was visualized using Principal Coordinates Analysis (PCoA) based on Bray – Curtis dissimilarity to assess differences in community structure across treatment groups. Variations in beta-diversity across different groups were statistically evaluated using permutational multivariate analysis of variance (PERMANOVA), implemented through the web-based analytical platform MicrobiomeAnalyst.^42^ The betadisper() test was applied using the R program package “VEGAN” and PERMANOVE was calculated using the package “VEGAN,” via the function adonis() to assess homogeneity of group dispersions. The dispersion did not significantly differ between groups, supporting the validity of our PERMANOVA results. Alpha diversity reflecting species richness and evenness within the microbial profiles was performed between different groups using Kruskal – Wallis test followed by pair-wise Mann – Whitney U comparison. A filtering threshold was applied to exclude taxa with relative abundance below 0.01% or low prevalence across samples to improve the reliability of downstream analyses. This preprocessing step was used in place of false discovery rate correction to control for noise in high-dimensional microbiome data and reduce the influence of spurious low-abundance features, as previously reported.^6,39^ The LEfSe was employed to identify distinct bacterial taxa contributing to differences among study groups with a logarithmic LDA score threshold set at 3, and the strategy for multiclass analysis was set to “all-against-all.”^43^ Paired Wilcoxon signed-rank tests were used to compare bacterial taxa before and after the intervention. Heatmap, volcano plot, and correlation analysis were performed using R v4.1 using the packages ggplot2, calibrate, Hmisc, and Corrplot packages in R v4.1.

For targeted metabolomics raw data was reported in micromoles using a reference standard, and data are presented as scaled and normalized change from baseline (change in mean divided by the standard deviation). Univariate analysis was used to analyze features independently, t-tests were performed on all features and groups of samples. For untargeted metabolomics, multivariate analysis was performed to compare relative contributions of independent variables to overall sample variance and separation between groups (Met and Met+Lira). To control for type 1 and 2 errors, we conducted multiple hypothesis testing correction via false discovery rate (Benjamin Hochberg), signal response evaluation, and calibration curve analysis.^44^ Statistical significance was based on *P*-value < 0.05 and log2 fold change of >1.

## Results

Table 1 illustrates the demographic and metabolic characteristics of the participants pre- and post-treatment. Most participants were female (60%), aged 15.4 ± 2.1 years, 24% were previously treated with metformin, and had Y-T2D for 1.3 ± 1.2 years. Participants self-reported standard diet and none were engaged in regular physical activity (>150 min/week). At baseline, demographic and metabolic characteristics were similar between groups, except hemoglobin A1c was ~1% higher in Pre-Met+Lira compared to Pre-Met (Suplementary S1).

**Table 1.** Demographic and metabolic characteristics at baseline and after intervention.

	Pre-Met (*n* = 14)	Post-Met	Pre-Met+Lira (*n* = 11)	Post-Met+Lira
Age (years)	16.3 (15.1, 17.4)		15.6 (14.3, 17.0)	
Female sex n (%)	8 (57)		7 (64)	
Duration of (years)	1.5 (0.8, 2.2)		1.0 (0.3, 1.7)	
Metformin naive n (%)	1 (7)		5 (45)	
Body Mass Index (kg/m^2^)	39.8 (35.0, 44.5)	41.1 (36.1, 46.0)	38.2 (32.8, 43.6)	37.3 (31.6, 42.9)
Systolic Blood Pressure (mmHg)	130.3 (122.6, 138.0)	127.8 (121.0, 134.6)	128.2 (118.6, 137.8)	122.1 (114.9, 129.3)
Hemoglobin A1c (mmol/mol)	46.3 (41.6, 50.9)	47.1 (39.4, 54.7)	57.6 (51.3, 63.8)	50.0 (34.2, 65.8)
Fasting glucose (mmol/L)	6.55 (5.72, 7.38)	5.94 (5.38, 6.55)	7.88 (6.11, 9.66)	6.6 (4.38, 8.82)
2-hour glucose (mmol/L)	13.2 (10.7, 15.7)	10.32 (8.71, 11.93)	15.2(11.9, 18.4)	9.99(6.77, 13.2)
Beta-cell function (sigma)	0.96 (0.13, 1.8)	0.99 (0.29, 1.7)	0.30 (0.06–0.53)	1.02 (0.51, 1.53)
Whole body insulin sensitivity (10^−4^ ml/microU/min)	0.15 (0.01, 0.29)	0.12 (0.07, 0.18)	0.14 (0.04, 0.24)	0.16 (0.07, 0.26)

Data are mean (95% CI), or n (%). Pre-Met: before metformin treatment, Post-Met: after metformin treatment, Pre-Met+Lira: before metformin and liraglutide treatment, Post-Met+Lira: after metformin and liraglutide treatment.

### Gut microbial profiles in untreated Y-T2D were diverse

**Figure 2.**
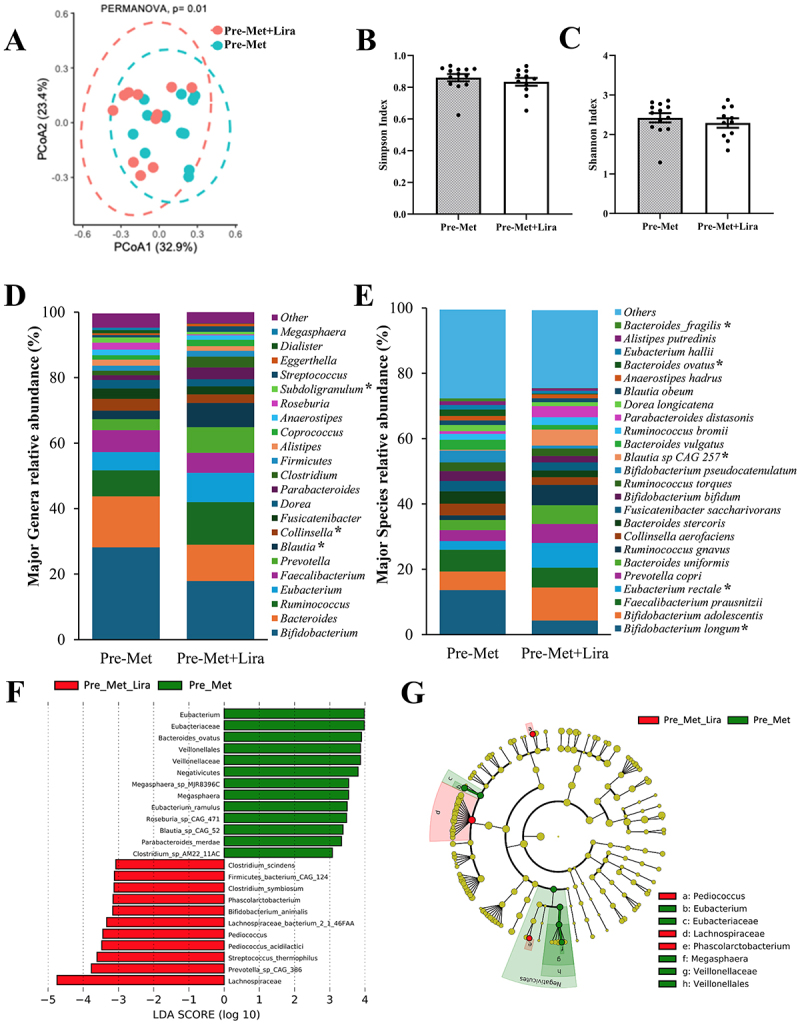
Differences in gut microbiome architecture at baseline in the metformin (Met) and metformin+Liraglutide (Met+lira) groups.

Using metagenomic sequencing of stool samples and advanced bioinformatic pipelines, we showed that β-diversity was significantly different (Figure 2(A)) but α diversity was not different by group (Figure 2(B,C), Chao1 data not shown). Pre-Met had a greater relative abundance of *Megasphaera*, *Subdoligranulum*, and *Collinsella* and a lower abundance of *Streptococcus* and *Blautia* (*p* < 0.05) (Figure 2(D)). Pre-Met was also associated with a higher relative abundance of *Bacteroides fragilis*, *Bacteroides ovatus, and Bifidobacterium longum*, but lower *Blautia sp*. and *Eubacterium rectales* compared to the Pre-Met+Lira (*p* < 0.05) (Figure 2(E)). The LEfSe identified significant bacterial taxa differences between the groups at baseline with notable differences at the genus and species level; Pre-Met was characterized by *Eubacterium, Bacteroides ovatus, Roseburia species*, and *Blautia species* (LDA = 3.0, Figure 2(F,G)).

(A) Principal Coordinates Analysis (PCoA) demonstrating significant differences in beta-diversity, assessed using the Bray-Curtis dissimilarity index, at baseline between Pre-Met (blue circles), *n* = 13 versus Pre-Met+Lira (pink circles), *n* = 11). (B-C) Bar charts and individual samples (black dots) of alpha diversity indices, Simpson and Shannon Index, at baseline for Pre-Met (gray bar) and Pre-Met+Lira (white bar). Data shown are mean and standard error of mean and compared with the Kruskal–Wallis test. Asterix (*) indicate significant differences between groups at *P* < 0.05. (D-E) Bar charts of the relative abundance of major genera and species differed at baseline between Pre-Met and Pre-Met+Lira, compared with Kruskal–Wallis test, *n* = 13. (F) LEfSe (Linear discriminant analysis Effect Size) plot and (G) cladogram representing significantly unique bacterial taxa in Pre-Met (green) versus Pre-Met+Lira (red) groups.

To determine the relationship of microbial abundance with glucose-insulin dynamics, we modeled glucose and insulin kinetics with the Insulin Sensitivity and Secretion (ISS) model during a frequently sampled 75 g two-hour oral glucose tolerance test^38^ and estimated post-absorptive rates of glucose appearance and gluconeogenesis with stable isotope tracers of glucose and deuterated water.^37^ Relative bacterial abundance did not correlate with whole-body insulin sensitivity, β-cell function, glucose rate of appearance, rates of gluconeogenesis, hemoglobin A1c, or metformin concentrations in either group (*p* > 0.1, data not shown). Baseline differences in microbial abundance were not associated with treatment response, measured as hemoglobin A1c or fasting glucose (*p* > 0.1).

### Metformin monotherapy changed gut microbiome signatures

**Figure 3.**
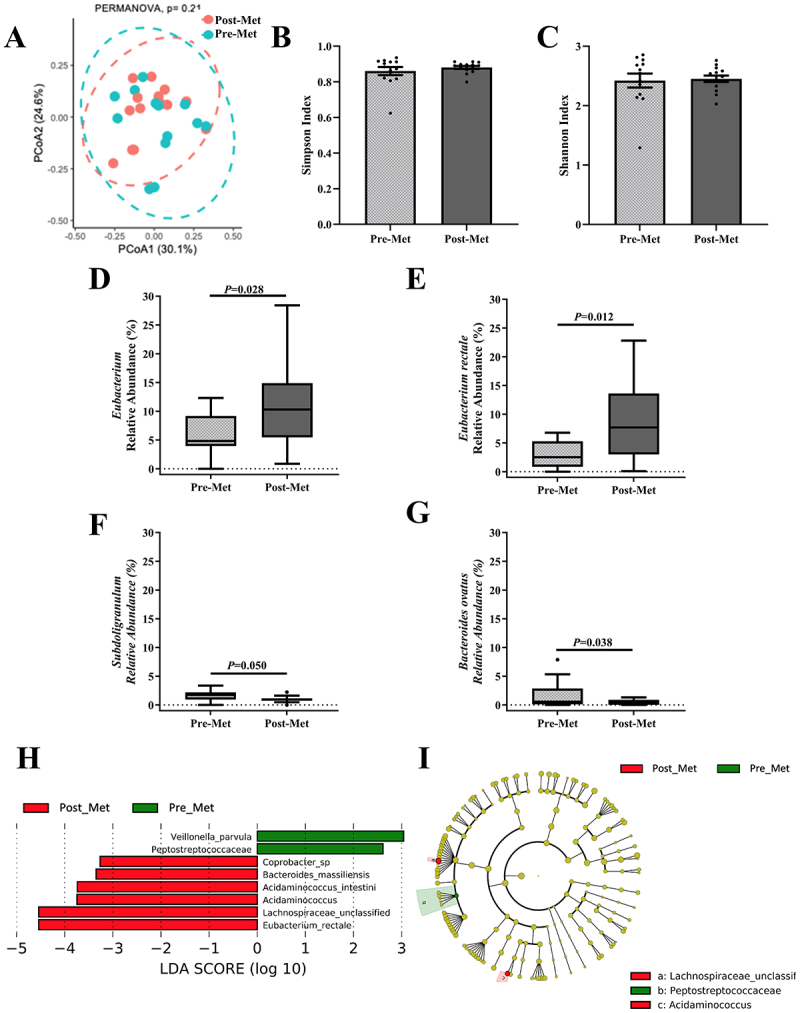
Changes in gut microbiome composition after metformin (Met) only.

To evaluate the effects of metformin monotherapy, we compared the changes in the gut microbiome composition and their relationship with glucose and insulin indices before and after treatment in the Met arm only. Met was not associated with significant changes in α or β diversity (Figure 3A–C). Met was associated with higher *Eubacterium* (Δ: 5.72%; 95% CI: 0.076, 11.36) and *Eubacterium rectale* (Δ: 5.81%; 95% CI: 1.21, 10.42) and lower *Subdulogranulum* (Δ: -0.74%; 95% CI: −1.4, −0.071) and *Bacteroides ovatus* (Δ: -1.26%; 95% CI: −2.79, 0.26) relative abundance (Figure 3D–G, Supplementary S6, *p* < 0.05). The LEfSe plot showed significant differences in abundance at the species level, including *Eubacterium rectale* (Figure 3H). Met modestly decreased two-hour glucose and tended to reduce fasting glucose (Table 1). Met-induced changes in microbial abundance were not associated with whole-body insulin sensitivity, β-cell function, hemoglobin A1c, fasting and two-hour glucose concentrations, glucose rate of appearance, gluconeogenesis, or metformin concentrations (*p* > 0.05, data not shown).

(A) Principal Coordinates Analysis (PCoA) indicating no change in β-diversity, assessed using the Bray-Curtis dissimilarity index, between Pre-Met (blue circles) versus Post-Met (pink circles), *n* = 13. (B-C) Bar charts and individual samples (black dots) of αdiversity indices, Simpson and Shannon Index, were not different between Pre-Met (light gray) versus Post-Met (dark gray), *n* = 13. Data shown are mean and standard error of mean and compared with the Kruskal–Wallis test. (D-E) Tukey box plots of relative abundance of genera *Eubacterium* and species *Eubacterium rectale* were significantly higher after metformin, compared with Wilcoxon test, *n* = 12. (F-G) Tukey box plots of relative abundance of genera *Subdoligranulum* and species *Bacteroides ovatus* were significantly lower after metformin, *n* = 12 (H) LEfSe (Linear discriminant analysis Effect Size) plot and (I) cladogram representing significantly unique bacterial taxa in Pre-Met versus Post-Met, *n* = 13. Statistical significance was determined as *P*≤0.05.

### Metformin and liraglutide induced distinct shifts in gut microbial composition

**Figure 4.**
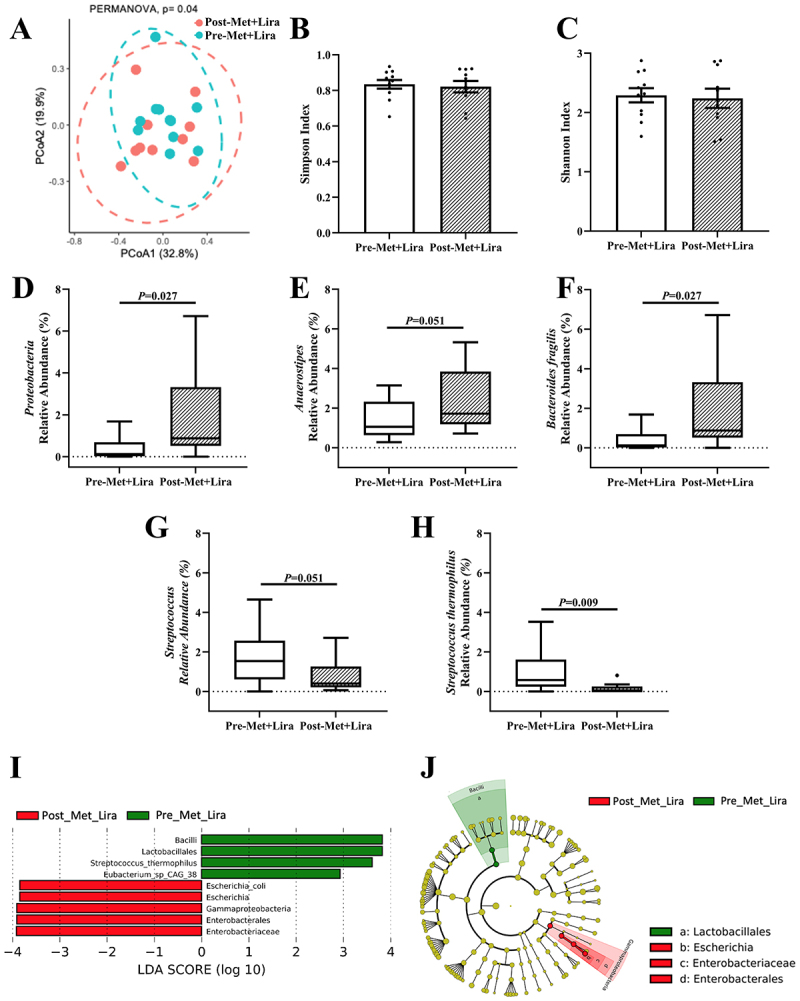
Changes in gut microbiome composition after metformin and liraglutide (Met+lira).

Next, we determined the effects of Met+Lira on the gut microbial composition. Three months of Met+Lira was associated with significant changes in β diversity (Figure 4A, *p = 0.04*) but was not associated with significant changes in α diversity (Figure 4B,C). Met+Lira did not change the Operational Taxonomic Unit (OTU) or *Firmicutes/Bacteroidetes* ratio (Supplementary Figure 7). Met+Lira was associated with higher abundance of phyla *Proteobacteria* (Δ: 1.54%; 95% CI: −0.065, 3.14), increased the relative abundance of genera *Anaerostipes* (Δ: 0.95%; 95% CI: −0.41, 2.31) and *Bacteroides fragilis* (Δ: 5.15%; 95% CI: −1.72, 12.01) and decreased genera *Streptococcus* (Δ: -0.97%; 95% CI: −2.14, 0.20) and species *Streptococcus thermophilus* (Δ: -0.86%; 95% CI: −1.68, −0.039) (Figure 4E–H, *p* ≤ 0.05). The LEfSe plot showed that Met+Lira was related to *Escherichia coli, Gammaproteobacteria, Enterobacterales*, and *Enterobacteriaceae* (Figure 4I,J). Met+Lira significantly reduced HbA1c and fasting and two-hour glucose, and increased β-cell function, but these changes were not associated with changes in gut microbiota relative abundance (Table 1).

(A) Principal Coordinates Analysis (PCoA) demonstrating significant differences in beta-diversity, assessed using the Bray-Curtis dissimilarity index, between Pre-Met+Lira, *n* = 11 versus Post-Met+Lira,*n* = 10. (B-C) Bar charts and individual samples (black dots) of alpha diversity indices, Simpson and Shannon Index, were similar between Pre-Met+Lira,*n* = 11 versus Post-Met+Lira, *n* = 9. Data shown are mean and standard error of mean and compared with the Kruskal–Wallis test. (D-F) Tukey box plots of relative abundance of phyla *Protebacteria*, genera *Anaerostipes*, and species *Bacteroides fragilis* were significantly higher after Pre-Met +Lira, compared with Wilcoxon test, *n* = 9. (G-H) Tukey box plots of relative abundance of genera *Streptococcus*, and species *Streptococcus fragilis* were significantly lower after Pre-Met +Lira, compared with Wilcoxon test, *n* = 9. (I) LEfSe (Linear discriminant analysis Effect Size) plot and (J) cladogram representing significantly unique bacterial taxa in Pre-Met+Lira versus Post-Met+Lira, *n* = 11. Statistical significance was determined as *P*≤0.05.

### Metformin upregulated prandial gut hormones

Since both Met and Met+Lira were associated with increased the relative abundance of SCFA-producing bacteria (including *Eubacterium rectale* and *Bacteroides fragilis*) we investigated compositional changes in the entero-insular pathway activation. Short-chain fatty acids should increase peptide YY (PYY) and incretin (glucagon-like peptide-1, [GLP-1] and gastrointestinal polypeptide [GIP]) hormone secretion via G-protein coupled receptors (GPR41 and GPR43).^23,24,45^ Using targeted metabolomics (nuclear magnetic resonance spectroscopy), we measured plasma SCFA and metabolite concentrations but found no changes post-intervention with Met or Met+Lira after corrections for multiple hypothesis testing (Suplementary Table 3). Incretin concentrations were measured at 0 and 120 min during the 2-h OGTT. Met increased postprandial PYY (Δ 1.67 (0.155–3.17) 95% CI) pM, *p* = 0.03, but did not change GLP-1 concentrations (Δ 1.05 (−1.07, 3.16), 95% CI) pM, *p* = 0.29 (Supplementary Table 2). Met+Lira increased postprandial PYY (Δ 2.31 (−0.0487–4.68) 95% CI) pM, *p* = 0.05 and fasting GIP (Δ 9.56 (0.406–18.7), 95% CI) pM, *p* = 0.04) (Supplementary Table 2). Endogenous GLP-1 concentrations after Met+Lira intervention could not be evaluated due to liraglutide assay cross-reactivity (see Supplementary Materials).

### Differential systemic pathway changes with metformin and liraglutide

**Figure 5.**
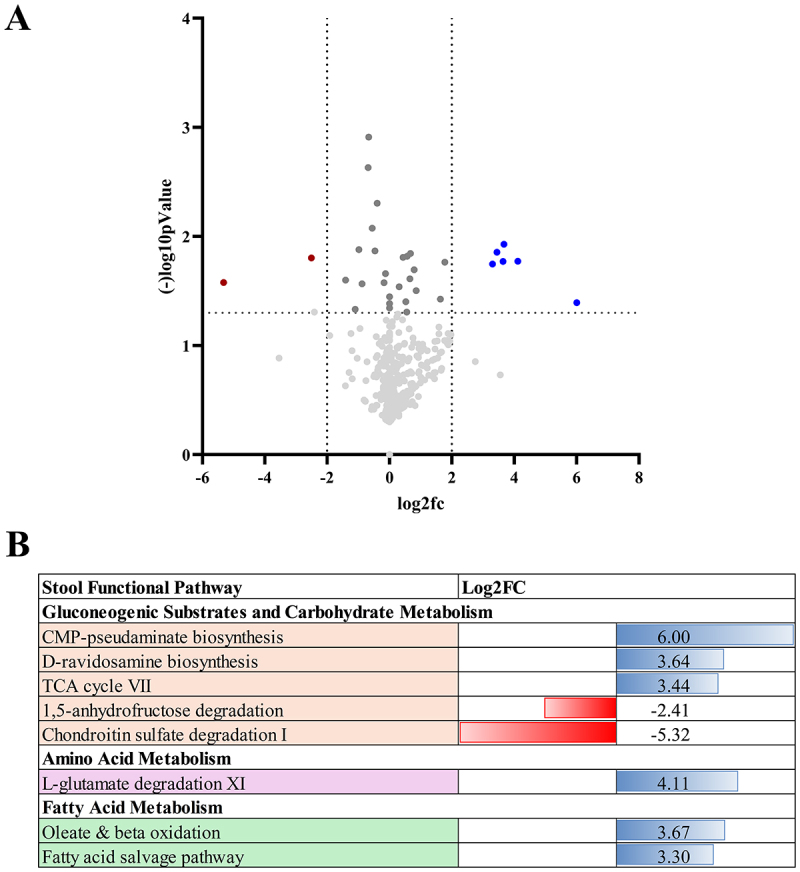
Differential expression of metabolic pathways after metformin (Met) only.

**Figure 6.**
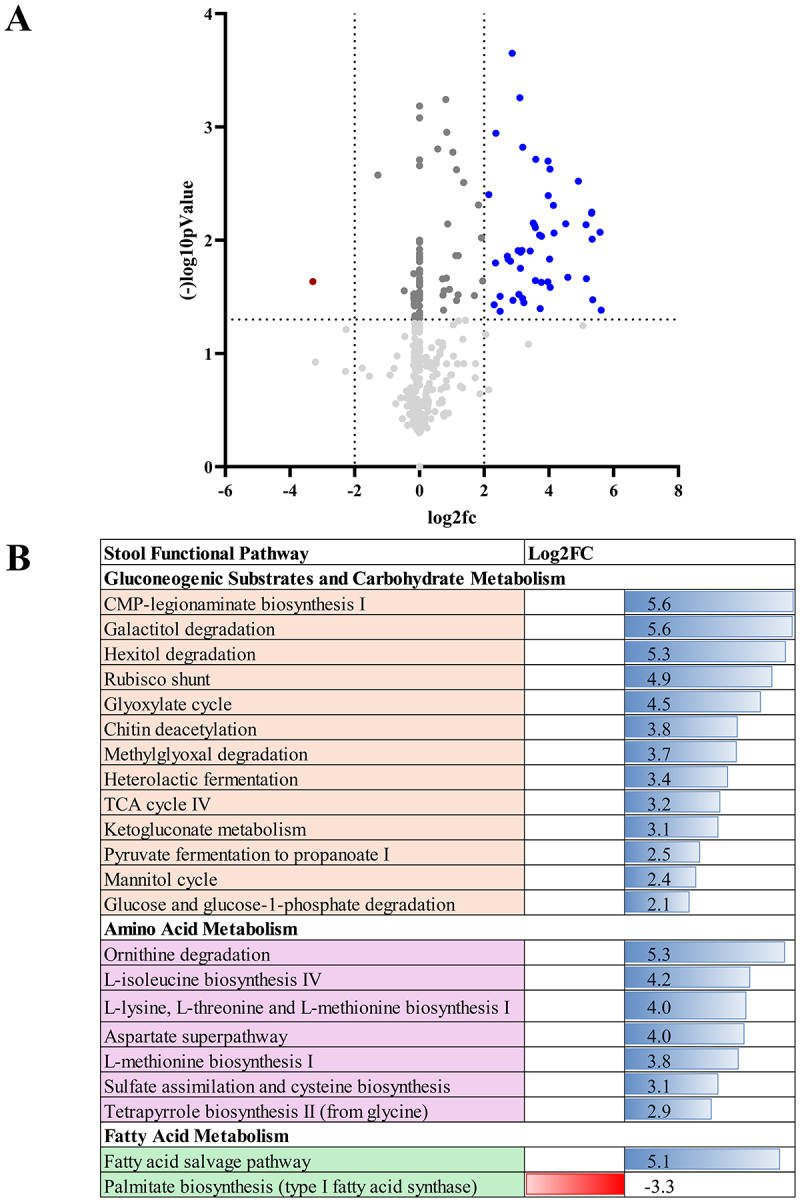
Differential expression of metabolic pathways after metformin and liraglutide (Met+lira).

Met was associated with downregulation of two and upregulation of six metabolic pathways , including the tri-carboxylic acid and beta-oxidation pathways, which could align with the increased abundance of SCFA bacteria (Figure 5). Met+Lira was associated with the upregulation of gluconeogenic substrates and multiple carbohydrate and amino acid pathways (*n* = 21) and downregulation one pathway of palmitate biosynthesis (Figure 6).

(A) Volcano plot of the change in functional profiling of stool microbiota after Met, as predicted by HuMANN, show metabolic pathways that are significantly upregulated (blue circles) and downregulated (red circles) in the stool microbiota. (B) Log fold change of metabolic pathways significantly upregulated (blue bars) and downregulated (red bars) after Met and categorized by metabolic pathway.

(A) Volcano plot of the change in functional profiling of stool microbiota Post-Met+Lira, as predicted by HuMANN, show metabolic pathways that are significantly upregulated (blue circles) and downregulated (red circles) in the stool microbiota. (B) Log fold change of metabolic pathways significantly upregulated (blue bars) and downregulated (red bars) Post-Met+Lira and categorized by metabolic pathway.

### Metformin upregulated bile acid deconjugating bacterial signatures and metabolism

The host microbiome is diverse and may act via multiple metabolic pathways to maintain glucose and insulin homeostasis. Bile acid metabolism has been identified as a critical metabolic mediator and signaling molecule.^46,47^ Therefore, we conducted untargeted metabolomics on fasting plasma samples before and after the intervention using ultra-high-performance liquid chromatography high-resolution tandem mass spectrometry (UHPLC-HRMS/MS).

**Figure 7.**
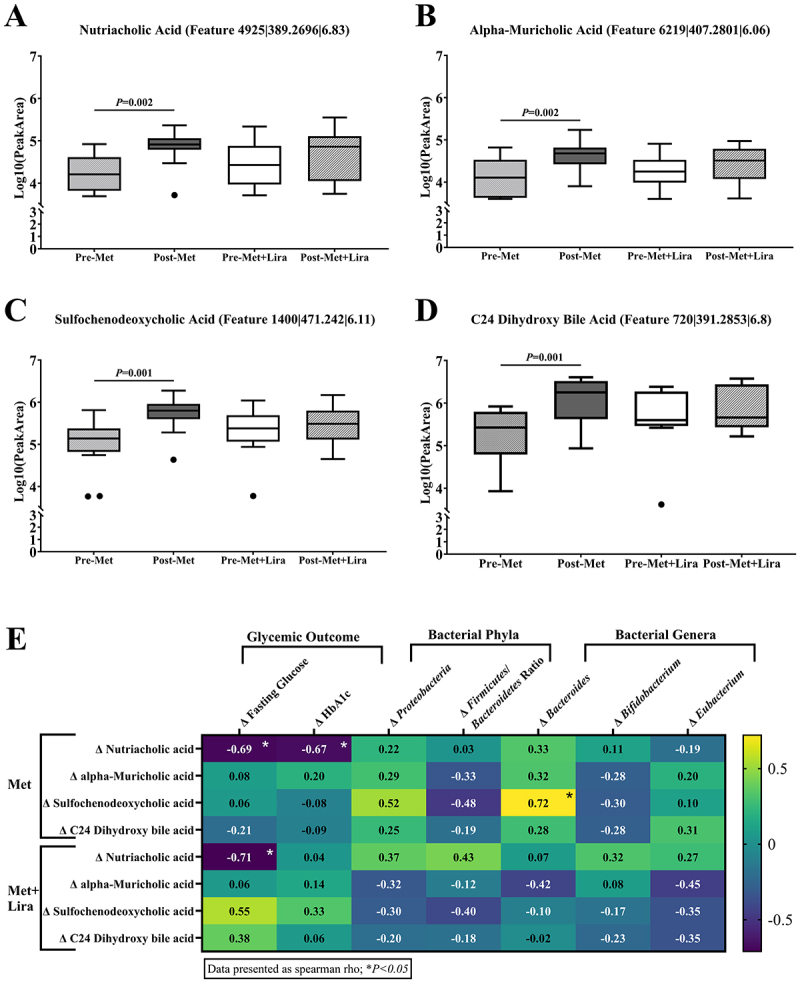
Change in secondary bile acid concentrations and relationship with gut microbial abundance and glycemia.

Met was associated with increase in all four of these secondary bile acids by 3–6-fold (*p* < 0.01), while the changes with Met+Lira were more variable (Figure 7A–D). The similarities in the increase in bile acids within the cholic acid class are shown in the similarities among the organic structure (Supplementary S5). Figure 7E illustrates the change in secondary bile acids with changes in glycemia and bacterial phyla and genera. Of the four secondary bile acids identified, the change in nutriacholic acid was significantly associated with reductions in fasting glucose (Met and Met+Lira) and HbA1c (Met only). Increased sulfochenodeoxycholic acid correlated with increased Δ *Bacteroides*, Δ *Firmicutes/Bacteroides ratio, and* Δ *Proteobacteria* in Met only *(p = 0.02)*. There were no correlations between the change in secondary bile acids and changes in bacterial species relative abundance (Supplementary Table 4).

**(A-D)** Tukey box plots of relative abundances of plasma secondary bile acids (measured as chromatographic peak area) found on untargeted metabolomic analyses. Metformin (Met) significantly increased nutriacholic, alpha-muricholic, sulfochenodeoxycholic, and C24 dihydroxy bile acid, but no significant changes were noted for metformin and liraglutide (Met+Lira). Secondary bile acid abundances were detected with ultra-high-performance liquid chromatography high-resolution tandem mass spectrometry and compared using the Kruskal–Wallis test followed by the Mann–Whitney U Test. **(E)** Heat map of association between change in plasma secondary bile acids by treatment group and change in glycemic outcome, stool bacterial phyla, and genera. Increased nutriacholic acid was associated with lower fasting glucose with Met and Met+Lira and lower hemoglobin A1c (HbA1c) in Met only. Increased sulfochenodeoxycholic acid was associated with greater relative abundance of phyla *Bacteroides* in Met only. Values are Spearman correlation coefficients (ρ) and asterisk (*) denote *P* < 0.05. P-values are corrected via multiple hypothesis testing (Benjamin Hochberg).

### Metformin and liraglutide changed host and gut-derived metabolites

To further characterize systemic effects of Met and Met+Lira on host metabolism, including ketone, gluconeogenic, and global amino acid pathways, we conducted targeted metabolomics of serum using nuclear magnetic resonance mass spectroscopy (NMR). Supplementary Table 3 shows the relative change in metabolites in both groups. As expected, Met and Met+Lira reduced glucose and mannose, while Met only increased the relative abundance of isoleucine, hydrophobic amino acids alanine and proline; however, values were no longer statistically significant after corrections for multiple hypothesis testing. There were no between-group differences in changes in metabolites (*p* ≥ 0.05).

Growing evidence in clinical and preclinical studies has also provided evidence for the cardioprotective effects of metformin therapy. Plasma Trimethylamine N-oxide (TMAO) – a gut-microbial derived cardiometabolic risk metabolite^48,49^—was measured with an enzyme-linked immunosorbent assay (ELISA). TMAO concentrations significantly decreased in the Met-only group (Δ: −0.54 (−2.69–0.02)) µM; median (25^th^−75^th^ percentile); *p* = 0.03), but no change was observed after Met+Lira (Δ: −0.52 (−1.88–1.65)) µM; median (25^th^−75^th^ percentile); *p* = 0.88). There was no correlation between change in TMAO concentrations and glycemia or bacterial composition (*p* > 0.05, data not shown).

## Discussion

Disentangling the effects of the gut microbiome on treatment responsiveness in Y-T2D has been challenging because few studies have examined the heterogeneity and complex interactions between the host, environment, and microbiota. This Y-T2D translational study sheds new light on the intestinal microbial architecture, metabolomics, and functional pathways to elucidate gut-based mechanisms of glycemic control of short-term metformin and liraglutide in African American Y-T2D under free-living dietary conditions. We showed that metformin therapy with and without liraglutide after 3 months was associated with an increased abundance of short-chain fatty acids and bile acid deconjugating bacteria, though gut microbial signatures were unique between the two medication groups. Upregulation of bile acid metabolites correlated with improvements in fasting glucose and overall glycemia. Since the increases in these secondary bile acids are known markers of bile acid deconjugation, these results suggest that activation of bile deconjugation pathways may be a potential mechanism of action for metformin in youth and could be key mediators of its systemic glucose-lowering effects.

Bile acid metabolism and signaling pathways modulate multiple nutrient pathways to maintain metabolic homeostasis and are critical intermediaries of drug mechanisms of action.^50,51^ We now show bile acid modulation as an important mediator of metformin and liraglutide mechanisms in youth, adding to the growing body of literature demonstrating metformin-induced upregulation of bile acid pathway in older adults.^52^ Increased plasma cholic secondary bile acid concentrations, byproducts from bacterial modification of primary bile acids, correlated with lower fasting glucose and hemoglobin A1c in both treatment arms, supporting their relationship with systemic metabolic effects. Both metformin alone and with liraglutide treatment arms increased bile-acid metabolizing microbiota, though robust correlations of changes in glycemia with bacterial OTUs were not detected and could be related to limited power to detect systemic changes. Microbial communities and bile salts interact bidirectionally within the intestinal lumen.^21,53^ Bacterial bile salt hydrolyzers are integral mediators of cholesterol metabolism and *de novo* bile acid synthesis.^54^ For example, *Eubacterium* hydrolyzes the C-24-N-acyl amide bond via bile salt hydrolase in conjugated bile acids to release glycine and taurine moieties.^54^ Downstream signaling cascades via FXR canonical bile acid receptors promote post-translational and post-transcriptional feedback regulation of nutrient density signal.^50^

Dynamic metformin-induced microbial changes also included greater abundance of SCFA-producing taxa – typically associated with healthier gut profiles – in alignment with published adult studies.^23,24,26,28,29^ The LefSe plot supported robust baseline differences in gut microbial signatures and a large effect size for up-regulation of butyrate-producing taxa with metformin with or without liraglutide. Metformin with or without liraglutide increased (*Eubacterium rectale, Bacteroides fragilis, and Anaerostipes*) and decreased relative abundance (*Bacteroides ovatus*) of different species of butyrate-producing bacteria and increased functional markers tri-carboxylic acid and beta-oxidation pathways. Other commonly reported SCFA-producing species upregulated by metformin include *Akkermansia muciniphila* and *Bifidobacterium adolescentis* species.^25^ Yet, numerical increases in one or two species may be less important compared to the changes in gut-related microbiome functional capacity. Differential and sometimes contradictory metformin-changes in species type and number reported across studies may be related to a variety of host and environmental factors.^24,55^ Bacterial taxa (number and composition) are influenced by host characteristics (hyperglycemia, age, genetics, diet, and activity levels), the environment (culture, geography), medications, and study design.^17,19,56^ This study design controlled for many of these confounders by recruiting adolescents with similar lifestyle behaviors and culture from localized geographic area.^35^ Our paired study design increased discrimination of host and treatment-specific changes across arange of SCFA genera and species for the first time in youth. By excluding youth with severe hyperglycemia (HbA1c > 9%), we also minimized hyperglycemic effects on the gut microbiome. Of note, between group differences in HbA1c at baseline were~0.7% but a specific gut microbial signature was not apparent for Met or Met+Lira. Altogether, our results demonstrate notable shifts toward butyrate-producing taxa in youth treated with metformin and liraglutide and underscored the importance of assessing baseline gut signatures to elucidate the interconnectivity and homeostasis of commensal gut microbiota in youth studies.

The mechanisms by which increased butyrate-producing organisms improve gut homeostasis in youth remain unclear. SCFA produced locally within the intestinal lumen may bind to G-protein coupled receptors (e.g. GPR-41) to stimulate incretin secretion.^57^ We observed increased PYY concentrations in both treatment arms consistent with activation of the entero-incretin axis demonstrated in prior studies.^58–60^ However, GLP-1 concentrations were not significantly increased in youth treated with metformin, and our observations do not confirm a direct mechanistic link. Contrary to our hypothesis, stool microbiota did not correlate with plasma SCFA concentrations or systemic glucose flux or metabolites. The volatility of SCFA resulting in low absolute plasma concentrations stretched the limits of detection for the current methodology, directly contributing to difficulties in discriminating entero-insular pathways from human *in vivo* studies. Additionally, SCFAs are primarily produced by colonic bacteria in the lower intestine with poor correlation between plasma levels and localized mucosal effects.^61^ Ideally, SCFA concentrations in the stool could have provided direct insight into microbiome-mediated changes, but these were not available in this study. Stool SCFA more accurately reflect local intestinal concentrations, whereas plasma SCFAs primarily represent absorbed and metabolically active fractions influenced by host utilization and clearance. In our study, only plasma samples were available for SCFA quantification, which limited our ability to assess local intestinal SCFA changes. Notably, increased SCFA stool concentrations may not predict serum changes as localized paracrine effect of stool SCFA within the intestinal milieu has been observed.^57^ Future studies including paired fecal and plasma measurements are needed to comprehensively understand SCFA dynamics and their role in metformin and liraglutide response and host health.

To further investigate localized activation of the entero-insulin axis, we performed microbial community functional profiling using the HMP Unified Metabolic Analysis Network (HUMNAnN) v2 pipeline, which estimates the metabolic and functional potential of the microbial populations. Pathway analysis identified differential regulation of pathways after 3 months of Met vs Met+Lira treatment. Both Met and Lira were associated with pathways of gluconeogenic substrates, carbohydrate metabolism, branch-chain amino acid, and tri-carboxylic acid cycling, highlighting the importance of the microbiome in maintaining energy metabolism (Figures 5 and 6).

Finally, this analysis was the first to characterize the gut microbiome in youth treated with combination of metformin and liraglutide to understand how combination therapy with a GLP-1 RA influenced metformin-induced gut microbial effects. Liraglutide monotherapy has changed host bacterial communities in mice^62^ and humans^9,32^; significantly increased intestinal bacterial diversity and richness by increasing the relative abundance of *Bacteroidetes*, *Proteobacteria*,^9^ and *Akkermansia*.^32^ We also demonstrated an increase in bile-acid deconjugating and SCFA-producing bacteria and increased Proteobacteria with Met+Lira. However, these changes could not be definitively attributed to metformin or liraglutide because of our study design. Downregulation of *Streptococcus* genera were also observed in Met+Lira group, however decreased concentrations of Streptococcus, could have been secondary to the synergistic or competitive effects of Met+Lira and require further study. Metformin monotherapy has previously been associated with increased abundance of Streptococcus genera.^9^ In contrast, liraglutide monotherapy did not increase Streptococcus abundance and was negatively associated an inflammatory marker, interleukin-6,^9^. Since the direct intestinal effects of liraglutide are implausible, further research is needed to determine whether the changes observed with Met+Lira were driven by metformin, liraglutide-related alterations in gastric emptying, or reciprocal gut microbial changes resulting from improved glycemic control and glucose-dependent insulin secretion. Additional studies could also investigate age-related changes in the microbiome with differential metformin responsiveness in youth compared to adults.

### Study strengths and limitations

The paired design accounted for intra-individual variability in an African American youth cohort, an underrepresented group in microbiome research. Additionally, deep phenotyping techniques were used to assess glucose and insulin dynamics and microbiome phenotyping. This two-drug analysis also used cutting-edge sequencing for metabolic and microbial analysis. By utilizing an untargeted metabolomic approach, a broad range of chemical changes were explored in an unbiased manner, allowing for the discovery of unexpected changes. The study’s small sample size, variable diabetes duration of participants, prior metformin use, and no diet standardization were important study limitations. Dietary intake is closely linked with individual gut microbial signatures.^11^ Prebiotic intake, with and without a standardized diet, markedly changed the relative bacterial abundance in Y-T2D.^6^ Although we attempted to mitigate these confounding effects by using a paired study design in Y-T2D who had similar dietary self-reported patterns during the study, future studies with standardized meals are necessary for isolating the individual microbiome-mediated drug responses. The limited sample and multiple comparisons in this study reduced statistical power, and significant correlations should be interpreted cautiously. Although considered an advantage for optimizing detection of microbial signatures, the homogeneous participant population limits generalizability and additional studies across diverse Y-T2D are warranted. It is also notable that despite randomization baseline HbA1c was higher in the Met+Lira group and analyses were adjusted accordingly. These factors could have contributed to greater regression to the mean homeostatic balance of the gut microbiota during this study intervention but would not explain the significant and consistent changes toward similar bacterial taxa in the both groups.

A recognized limitation of our microbial analyses was the lack of methodological cross-validation using alternative statistical tools such as DESeq2, ANCOM, or ALDEx2. These tools address the compositional constraints and dispersion characteristics of microbiome sequencing data, though no gold standards exist.^63^ Reducing the false discovery rates is critical and is balanced with identifying meaningful biological signals. Consequently, LEfSe analyses adeptly identified differentially abundant taxa, but Lefse methodology is sensitive to false positives because it assumes independence between features and does not explicitly model the compositional nature of microbial communities. To minimize the false discovery rate, we used a filtering threshold which limited our ability to detect subtle community shifts, particularly in low-abundance taxa or under conditions of high inter-individual variability. Our decision to use LEfSe was guided by its widespread adoption in microbiome research and its suitability for exploratory analyses that aim to reveal microbial signatures with biological relevance. Parallel analyses with DESeq2 (which incorporates variance stabilization and shrinkage estimation), ANCOM (which accounts for compositionality through log-ratio transformations), or ALDEx2 (which uses Monte Carlo sampling from a Dirichlet distribution) could improve the robustness and reproducibility of our findings but were not available for this study. Future investigations would benefit from a multi-method approach to differential abundance testing, allowing for triangulation of results across statistical paradigms to enhance the resolution of subtle ecological changes within complex microbial ecosystems. Detailed phenotyping with next-generation tools will be ideal for characterizing age-related changes in gut microbiota diversity. Future studies comparing youth and adults with type 2 diabetes could leverage these tools to further determine whether dynamic changes in Y-T2D microbial abundance explain variations in metformin response between Y-T2D and adults with type 2 diabetes.

## Conclusions

Short-term metformin with or without liraglutide in Y-T2D induced distinct shifts toward short-chain fatty acid producing and bile acid deconjugating gut microbiota taxa. Increased secondary bile-acid metabolites correlated with improved fasting glycemia, suggesting a central role for bile acid metabolism in mediating systemic microbiome effects. Future studies are needed to investigate the mechanistic pathways underlying metformin therapy in a diet-controlled environment with a larger sample size.

## Supplementary Material

mighty revision supplement 2025AUG24.docx

## Data availibility

The data presented in the study are deposited in BioProject repository, accession number PRJNA1281554; https://dataview.ncbi.nlm.nih.gov/object/PRJNA1281554?reviewer=l8fjfb4ithslelmk1a4v16m8n8.
