# Supplementary material for: Systemic and gut microbiome changes with metformin and liraglutide in youth-onset type 2 diabetes: the MIGHTY study
Source: Gut Microbes. 2025 Sep 29;17(1):2558071. doi: 10.1080/19490976.2025.2558071 (PMC12482426; doi:10.1080/19490976.2025.2558071)
Supplement: mighty revision supplement 2025AUG24.docx [file KGMI_A_2558071_SM3064.docx]

**Systemic and Gut Microbiome Changes with Metformin and Liraglutide in Youth-Onset Type 2 Diabetes: The MIGHTY Study**

**Supplementary Methods**

Contents

[Clinical Study Protocol 1](#_Toc206771114)

[Study Agents/ Intervention 1](#_Toc206771115)

[Dose titration for gastrointestinal intolerance 2](#_Toc206771116)

[Metagenomic Shotgun sequencing 3](#_Toc206771117)

[Untargeted Metabolomics 3](#_Toc206771118)

[Untargeted metabolomics data processing 6](#_Toc206771119)

[Measurement of Glucagon like-1 peptide (GLP-1) concentrations: 7](#_Toc206771120)

[Supplemental Tables 8](#_Toc206771121)

[Supplementary Table 1: Baseline demographic and metabolic characteristics. 8](#_Toc206771122)

[Supplementary Table 2: Change in incretin concentrations after intervention. 9](#_Toc206771123)

[Supplementary Table 3: Change in Plasma Metabolomic Profile After Intervention 10](#_Toc206771124)

[Supplementary Table 4: Correlation of change in bile acid concentrations with glycemia and bacterial taxa by intervention group. 12](#_Toc206771125)

[Supplemental Figures 13](#_Toc206771126)

[Supplementary Figure 1. Participant flow diagram 13](#_Toc206771127)

[Supplementary Figure 2. Nutriacholic Acid (Feature 4925|389.2696|6.83) chromatographic retention time compared against standards with the same exact mass (m/z) to compare retention times. 14](#_Toc206771128)

[Supplementary Figure 3. Alpha-Muricholic Acid (Feature 6219|407.2801|6.06) chromatographic retention time compared against standards with the same exact mass (m/z) to compare retention times. 15](#_Toc206771129)

[Supplementary Figure 4. C24 dihydroxy bile acid (Feature 720|391.2853|6.8) chromatographic retention time compared against standards with the same exact mass (m/z) to compare retention times. 16](#_Toc206771130)

[Supplementary Figure 5. Cholic secondary bile acid organic structures. 17](#_Toc206771131)

[Supplemental Figure 6: Association of Baseline Microbial Abundance with Metabolic Variables 18](#_Toc206771132)

[Supplementary Figure 7. Microbiome signatures before (Pre-Met) and after metformin (Post-Met). 19](#_Toc206771133)

[Supplementary Figure 8. Microbiome signatures before (Pre-Met+Lira) and after metformin and liraglutide (Post-Met+Lira). 20](#_Toc206771134)

[References 22](#_Toc206771135)

# Clinical Study Protocol

## Study Agents/ Intervention

Two study agents were used: metformin oral 500mg oral tablet and liraglutide (6mg/ml, 3ml) solution for subcutaneous injection, pre-filled, multi-dose pen that delivered doses of 0.6mg, 1.2mg or 1.8mg. Both metformin and liraglutide were used within the approved dosing regimens. Neither drug was altered from the approved dosage formulation. Table 3 illustrates the titration schedule for each study drug.

**Table : Titration Schedule for Metformin and Liraglutide**

| Study schedule | Week | Liraglutide  (subcutaneous injection) | Metformin  (oral tablet) |
| --- | --- | --- | --- |
| Run-in | -1 to 0 | - | - |
| After baseline visit | 0-1 | 0.6mg once daily | 500mg once daily |
|  | 1-2 | 1.2mg once daily | 500mg twice daily |
| **Target study dose** | 3-12 | 1.8mg once daily | 1000mg twice daily |

The participant started the study drug(s) on Day 2 of Visit 2, after the tracer protocol was completed. The PI reviewed blood glucose logs and/ or CGM readings weekly and ensured that the study drug(s) were titrated to the highest tolerable dose as guided by Table 3 above. For patients on combination therapy, the dose of liraglutide were not increased if the fasting blood glucose was <75mg/dl on 2 or more consecutive days. If the fasting blood glucose subsequently rose to ≥90mg/dl on 2 or more consecutive days, the dose of liraglutide was increased by 0.6mg once daily every 5-7 days to achieve maximum tolerable dose.

## Dose titration for gastrointestinal intolerance

For participants on the combination therapy arm who experienced gastrointestinal side effects, the dose of liraglutide was decreased first. Liraglutide dosage was decreased by 0.6mg once daily. If gastrointestinal symptoms persisted after 2-3 days, metformin dose was decreased by 500mg. If symptoms persist, dose adjustments were made every 2-3 days, alternating between liraglutide (0.6mg) and metformin (500mg) dose reductions. If the highest tolerable metformin dose was < 1000mg daily or liraglutide <0.6mg daily, the subject was withdrawn from the study.

For participants on metformin alone, metformin was decreased by 500mg every 2-3 days until symptoms resolved. If the highest tolerable dose of metformin was <1000mg daily, the subject was withdrawn from the study.

If gastrointestinal symptoms resolved, and at the discretion of the PI, the dose of metformin and/or liraglutide was increased every 2-3 days to the maximum tolerable dose. Incremental dose increases were as follows: metformin 500mg every 2-3 days and liraglutide 0.6mg every 2-3 days (PMID: 37967247 and DOI: 10.6084/m9.figshare.24492187).

# Metagenomic Shotgun sequencing

The analysis of shotgun sequencing data was conducted utilizing the Yet Another Metagenomic Pipeline (YAMP) workflow. The YAMP pipeline integrates tools from bbmap suite for sequence de-duplication, quality trimming, and decontamination [37]. FastQC was employed for the visualization of both raw and QC filtered metagenomic reads. MetaPhlAn (v3.0), a marker gene–based taxonomic profiler, was employed to characterize microbial community composition from metagenomic sequencing data. Unlike approaches relying on alignment of all sequencing reads, MetaPhlAn maps reads to a curated database of clade-specific marker genes, enabling high-resolution taxonomic profiling with reduced computational demand and lower false-positive rates. Relative abundances of taxa are calculated based on the proportion of reads mapping to these unique markers, a strategy that minimizes biases introduced by genome size variation or gene copy number differences. Because this method relies exclusively on informative marker genes rather than the complete genomic dataset, it does not provide absolute read counts per taxon instead, normalized relative abundance profiles form the basis for downstream statistical analyses in this study. References to the above type of analysis are PMID: 33944776 and PMID: 22434009.

# Untargeted Metabolomics

**Sample Extraction**

Samples were prepared by protein precipitation with cold organic solvent. 800 microliters of ice-cold methanol (Fisher Chemical, Optima™ LC-MS grade) were added to 200 microliters of plasma each in a 1.5 mL microcentrifuge tube. The samples were vortexed briefly (10 s) and placed in a –20°C freezer for 30 min to facilitate precipitation. Samples were subsequently centrifuged for 10 minutes at 14000 relative centrifugal force (rcf) at 4°C (Eppendorf Centrifuge 5425R). 800 microliters of supernatant, referred to as the extract, were transferred into a new 1.5 mL microcentrifuge tube and the pellet was discarded in accordance with chemical and biological safety procedures. The extract was completely dried via centrifugal evaporation (Genevac EZ-2 Plus) using the HPLC fraction setting and a maximum temperature of 40°C. The dried extracts were capped and stored at -80°C until prepared for analysis.

**Sample resuspension**

Dried extracts were removed from storage at -80°C, allowed to warm to room temperature, and resuspended via the addition of 80 microliters of water-acetonitrile 98%:2% v/v. Resuspended extracts were briefly vortexed (15 s). An aliquot from each extract was transferred into a new 1.5 mL microcentrifuge tube to create a pooled quality control sample. An additional aliquot was transferred to a 2 mL autosampler vial with microvolume insert (Agilent).

**Analytical measurement**

Samples were analyzed using an ultra-high performance liquid chromatograph (Vanquish, Thermo Scientific) coupled to a high-resolution mass spectrometer (Orbitrap Fusion Tribrid, Thermo Scientific). LC-MS and LC-MS/MS data were acquired. LC-MS data were collected from individual samples (n = 1 injection), system blanks (injection of solvent used to resolubilize samples), and a pooled quality control. The pooled quality control (QC) was injected multiple times at different volumes and used in data processing. LC-MS/MS data, used to annotate features, were collected using the AcquireX (Thermo Scientific) deep scan methodology in which pooled QC was injected multiple times (n = 7). Prior to measurement the mass spectrometer was calibrated using FlexMix (Thermo Scientific) following manufacture directions. Prior to sample acquisition, UHPLC-HRMS instruments were calibrated according to vendor benchmarks (Thermo Scientific Pierce FlexMix Calibration Solution). A system suitability standard containing a mixture of known analytes was routinely run throughout sample acquisition to evaluate instrument performance, reproducibility, and precision.

Chromatographic separation was carried out on a 2.1 x 100 mm, 100Å, 2.6 μm, F5 analytical column (Phenomenex) with corresponding guard cartridge. The column was maintained at 30°C during separation with solvent pre-heater. Gradient elution was performed after an initial period of isocratic elution using water with 0.1% acetic acid v/v (A) and acetonitrile with 0.1% acetic acid v/v (B). Separation was performed as follows: 0% B from 0 - 2.0 min, 0% to 100% B from 2.0 to 10.5 min, 100% B from 10.5 to 12.0 min, 100% to 0% B from 12.0 to 13.0 min, 0% B from 13.0 to 20.0 min. The flow rate was 0.5 mL min-1.

Ionization was performed via heated electrospray ionization (NG Ion Max, Thermo Scientific). The source parameters in positive ionization mode were as follows: spray voltage of +4000 V, sheath gas of 50 arbitrary units (arb), auxiliary gas of 10 arb, sweep gas of 1 arb, ion transfer tube at 325°C, vaporizer at 350 °C. The source parameters used in negative ionization mode were identical except for the spray voltage of -3000 V.

MS and MS/MS data were collected with an anticipated LC peak width of 8 s and a default charge of 1. EASY-IC™ (Thermo Scientific) was installed and used during data collection; a lock-mass is measured, concurrently to experimental measurement, and used for instrument mass calibration. MS data were acquired at 120,000 resolution from m/z 100-1000 with an RF lens of 60% and maximum injection time of 50 ms. MS/MS data were acquired at 30,000 resolution using an isolation width of 1.5 (m/z), stepped assisted HCD (energy steps were 20, 35, and 60, and a maximum injection time of 54 ms. The inclusion list was generated and updated via AcquireX with a low and high mass tolerance of 5 ppm. An intensity filter was applied via the “Intensity” node in the workflow with an intensity threshold of 2.0e4. The “Dynamic Exclusion” node was used with the following parameters: exclude after n = 3 times, if occurs within 15 s, exclusion duration of 6 s, a low mass tolerance of 5 ppm, a high mass tolerance of 5 ppm, and excluding isotopes.

# Untargeted metabolomics data processing

Compound Discoverer 3.3.0.550 (Thermo Scientific) was used to process.raw files which resulted in a tabular output which included descriptors of each feature (e.g. m/z, retention time), annotation information (e.g. MS/MS database match), and peak area. We processed the output from Compound Discoverer using in-house R scripts via JupyterNotebooks. The major components of the processing included formatting of the data outputs, comparison of m/z and retention time of annotation features versus an in-house generated list based on authentic chemical standards, assessment of signal response in pooled QC samples, assessment of signal variance in pooled QC samples versus samples (i.e. dispersion ratio), and multi- and univariate statistics. MSI levels of annotation confidence[1] were provided based on the MS/MS database matching algorithm in Compound Discoverer, a list of m/z generated from authentic chemical standards, and manual annotation. All features with a MS/MS database match remained at level 2 while those matching the m/z and retention time criteria were promoted to level 1.

# Measurement of Glucagon like-1 peptide (GLP-1) concentrations:

All samples were collected with protease inhibitor and 10µl of DPPIV inhibitor (4mM) per 1 ml of whole blood was added.  The inhibitor is present in the blood draw tube. Endogenous GLP-1 concentrations after Met+Lira intervention could not be evaluated, because of cross-reactivity of the assay with liraglutide. We measured active GLP-1 using the Meso Scale assay kit (Rockville, MD, Catalog number K1503OD) in plasma containing protease inhibitors and DPP4 inhibitor. To determine if Liraglutide cross-reacted with the assay, we spiked with increasing concentrations of Liraglutide. Increasing Liraglutide spike resulted in non-linear recovery of active GLP-1 measurements thus rendering us unable to use this assay kit. We also tested the active GLP-1 ELISA kit from Millipore- Sigma (Millipore-Sigma Catalog # EGLP-35K) and found similar results.

# Supplemental Tables

## Supplementary Table 1: Baseline demographic and metabolic characteristics.

|  | **Met (n=14)** | **Met+Lira (n=11)** | ***P*-value** |
| --- | --- | --- | --- |
| **Demographic** | | | |
| Age (years) | 16.3 ± 2.0 | 15.6 ± 2.0 | 0.23 |
| Female sex n (%) | 8 (57) | 7 (64) | 0.75 |
| Time since diabetes diagnosis (years) | 1.5 ± 1.2 | 1.0 ± 1.0 | 0.35 |
| Metformin naive n (%) | 1 (7) | 5 (45) | 0.03 |
| Body Mass Index (kg/m^2^) | 39.8 ± 8.2 | 38.2 ± 8.0 | 0.63 |
| Systolic Blood Pressure (mmHg) | 130.3 ± 13.4 | 128.2 ± 14.3 | 0.71 |
| Hemoglobin A1c (mmol/mol) | 46.3 ± 8.1 | 57.6 ± 9.3 | 0.003 |
| Fasting glucose (mmol/L) | 6.6 ± 1.5 | 7.9 ± 2.7 | 0.13 |
| 2-hour glucose (mg/dL) | 13.2 ± 4.1 | 15.2 ± 4.88 | 0.29 |

Data are mean±SD or n (%).

## Supplementary Table 2: Change in incretin concentrations after intervention.

| Hormone |  | Met | P-Value | Met+Lira | P-Value |
| --- | --- | --- | --- | --- | --- |
| Δ Peptide YY (PYY) (pM) | Fasting | 0.912  (-1.94- 3.76)* | *P=0.49* | 2.58  (0.965 – 6.12) | *P=0.14* |
|  | 2-Hour | 1.67  (0.155 - 3.17) | *P=0.03* | 2.31  (-0.0487 – 4.68) | *P=0.05* |
| Δ Gastric inhibitory polypeptide (GIP) (pM) | Fasting | -8.49  (-34.9 – 17.9)*** | *P=0.48* | 9.56  (0.406 – 18.7) | *P=0.04* |
|  | 2-Hour | -5.16  (-16.3 – 5.94)** | *P=0.32* | -2.85  (-12.7 – 6.96) | *P=0.53* |
| Δ Glucagon-Like Peptide-1  (GLP-1) pM | Fasting | 0.43  (-1.20 - 2.06)*** | *P=0.55* |  | |
|  | 2-Hour | 1.05  (-1.07 - 3.16)** | *P=0.29* |  |  |

Data are mean (95% CI). Met (n=11) and Met+Lira (n=11). Data was unable to be collected from some participants (^*^n=10; **n=9; ***n=8). Δ: Post-intervention – Pre-intervention.

## Supplementary Table 3: Change in Plasma Metabolomic Profile After Intervention

Changes in plasma metabolites from post- to pre-intervention with metformin only (MET) and metformin and liraglutide (MET+LIRA) across four metabolic pathways – short-chain fatty acid and ketone metabolism (yellow), gluconeogenic substrates and carbohydrate metabolism (orange), amino acid metabolism (purple), and alcohol metabolism (pink). Blue bars represent increases in metabolite concentration and red bars represent decreases in metabolite concentration. Data are scaled and normalized, expressed as the change in concentration (post-intervention minus pre-intervention) divided by standard deviation (Δ/σ) and delta (Δ) mean (95% Confidence Interval). Groups were compared with t-tests and unadjusted P-values presented. P-values<0.05 are highlighted in red. None of confidence intervals showed changes greater than 2 sigma and no significant differences were found after corrections for multiple hypothesis testing (Benjamin Hochberg).

## Supplementary Table 4: Correlation of change in bile acid concentrations with glycemia and bacterial taxa by intervention group.

| **Treatment Group** | **Bile Acid** | **Glycemic Outcome** | | **Bacterial Phyla** | | **Bacterial Genera** | | | **Bacterial Species** | | | |
| --- | --- | --- | --- | --- | --- | --- | --- | --- | --- | --- | --- | --- |
|  |  | Δ Fasting Glucose | Δ HbA1c | *Δ Proteobacteria* | *Δ Firmicutes/*  *Bacteroidetes Ratio* | *Δ Bacteroides* | *Δ Bifidobacterium* | *Δ Eubacterium* | *Δ Bacteroides Ovatus* | *Δ Bifidobacterium Adolescentis* | *Δ Bifidobacterium Longum* | *Δ Eubacterium Rectale* |
| **Met Only** | **Δ Nutriacholic Acid** | -0.69  (p=0.02) | -0.67  (P=0.02) | 0.22  (p=0.51) | 0.03  (p=0.94) | 0.33  (p=0.32) | 0.11  (p=0.75) | -0.19  (p=0.57) | 0.35  (p=0.29) | 0.35  (p=0.30) | -0.31  (p=0.35) | -0.15  (p=0.67) |
|  | **Δ alpha-Muricholic Acid** | 0.08  (p=0.81) | 0.20  (p=0.56) | 0.29  (p=0.39) | -0.33  (p=0.32) | 0.32  (p=0.34) | -0.28  (p=0.40) | 0.20  (p=0.55) | 0.20  (p=0.55) | -0.34  (p=0.31) | 0.07  (p=0.83) | 0.32  (p=0.34) |
|  | **Δ Sulfochenodeoxycholic Acid** | 0.06  (p=0.87) | -0.08  (p=0.83) | 0.52  (p=0.10) | -0.48  (p=0.13) | 0.72  (p=0.02) | -0.30  (p=0.36) | 0.10  (p=0.77) | 0.42  (p=0.20) | -0.46  (p=0.15) | -0.02  (p=0.96) | 0.25  (p=0.46) |
|  | **Δ C24 dihydroxy bile acid** | -0.21  (p=0.53) | -0.09  (p=0.80) | 0.25  (p=0.45) | -0.19  (p=0.57) | 0.28  (p=0.40) | -0.28  (p=0.40) | 0.31  (p=0.35) | 0.32  (p=0.34) | -0.02  (p=0.96) | -0.30  (p=0.36) | 0.42  (p=0.20) |
| **Met+Lira** | **Δ Nutriacholic Acid** | -0.71  (p=0.03) | 0.04  (p= 0.91) | 0.37  (p=0.34) | 0.43  (p=0.24) | 0.07  (p=0.86) | 0.32  (p=0.40) | 0.27  (p=0.48) | -0.25  (p=0.51) | 0.47  (p=0.20) | -0.07  (p=0.86) | 0.33  (p=0.37) |
|  | **Δ alpha-Muricholic Acid** | 0.06  (p=0.88) | 0.14  (p= 0.71) | -0.32  (p=0.40) | -0.12  (p=0.76) | -0.42  (p=0.26) | 0.08  (p=0.83) | -0.45  (p=0.22) | 0.23  (p=0.54) | -0.23  (p=0.55) | 0.18  (p=0.65) | -0.44  (p=0.23) |
|  | **ΔSulfochenodeoxycholic Acid** | 0.55  (p=0.12) | 0.33  (p= 0.38) | -0.30  (p=0.43) | -0.40  (p=0.28) | -0.10  (p=0.79) | -0.17  (p=0.66) | -0.35  (p=0.35) | 0.65  (p=0.06) | -0.14  (p=0.71) | -0.29  (p=0.44) | -0.36  (p=0.34) |
|  | **Δ C24 dihydroxy bile acid** | 0.38  (p=0.30) | 0.06  (p= 0.87) | -0.20  (p=0.60) | -0.18  (p=0.63) | -0.02  (p=0.97) | -0.23  (p=0.54) | -0.35  (p=0.35) | 0.63  (p=0.07) | -0.44  (p=0.23) | 0.21  (p=0.58) | -0.34  (p=0.36) |

Data are spearman rho (ρ) (P-value), Δ: Post-intervention – Pre-intervention.

# Supplemental Figures

## Supplementary Figure 1. Participant flow diagram





## Supplementary Figure 2. Nutriacholic Acid (Feature 4925|389.2696|6.83) chromatographic retention time compared against standards with the same exact mass (m/z) to compare retention times.

**
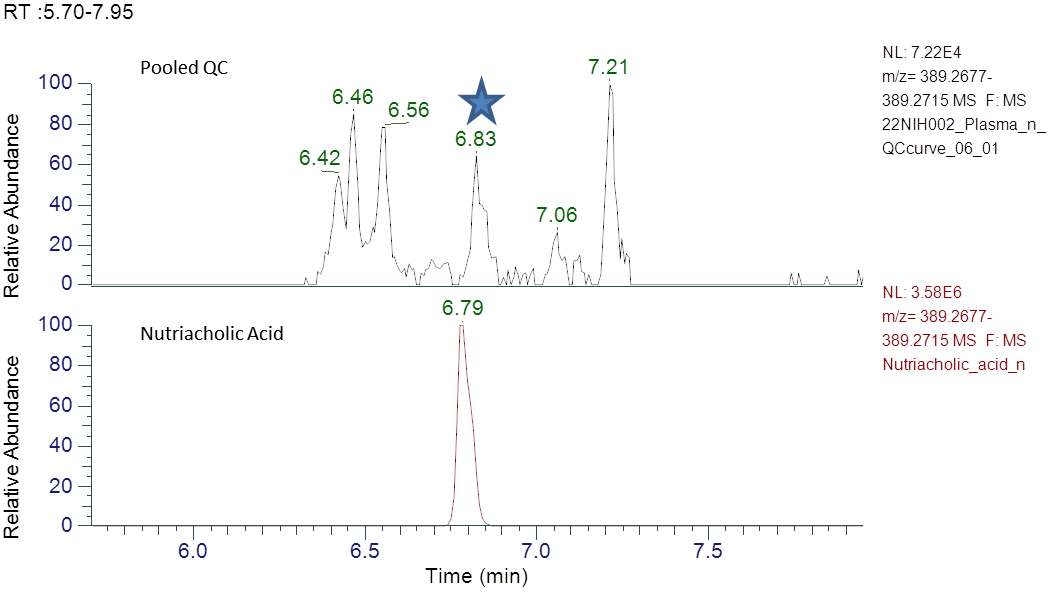
**

Chromatographic retention time of each in-house annotated bile acid feature (shown in pooled QC, top row) compared to chromatographic retention time of possible mass matches in in-house reference standard library. Annotations were assigned as the analyte with the closest retention time match, and the significant analyte peak annotated with a star.

## Supplementary Figure 3. Alpha-Muricholic Acid (Feature 6219|407.2801|6.06) chromatographic retention time compared against standards with the same exact mass (m/z) to compare retention times.

**
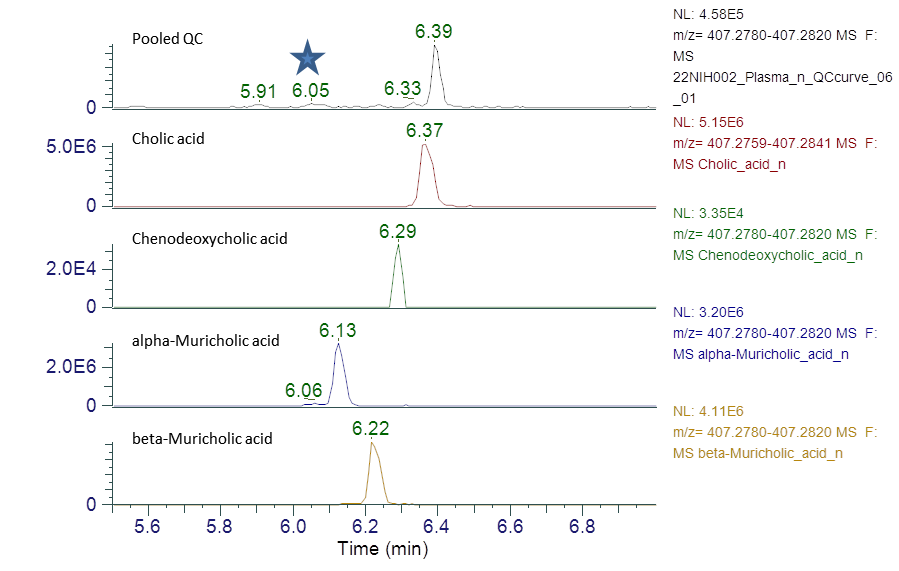
**

Chromatographic retention time of each in-house annotated bile acid feature (shown in pooled QC, top row) compared to chromatographic retention time of possible mass matches to in-house reference standard library (cholic acid, chenodeoxycholic acid, alpha-Muricholic acid, and beta-Muricholic acid). Annotations were assigned as the analyte with the closest retention time match (alpha-Muricholic acid), and the significant analyte peak annotated with a star.

## Supplementary Figure 4. C24 dihydroxy bile acid (Feature 720|391.2853|6.8) chromatographic retention time compared against standards with the same exact mass (m/z) to compare retention times.

**
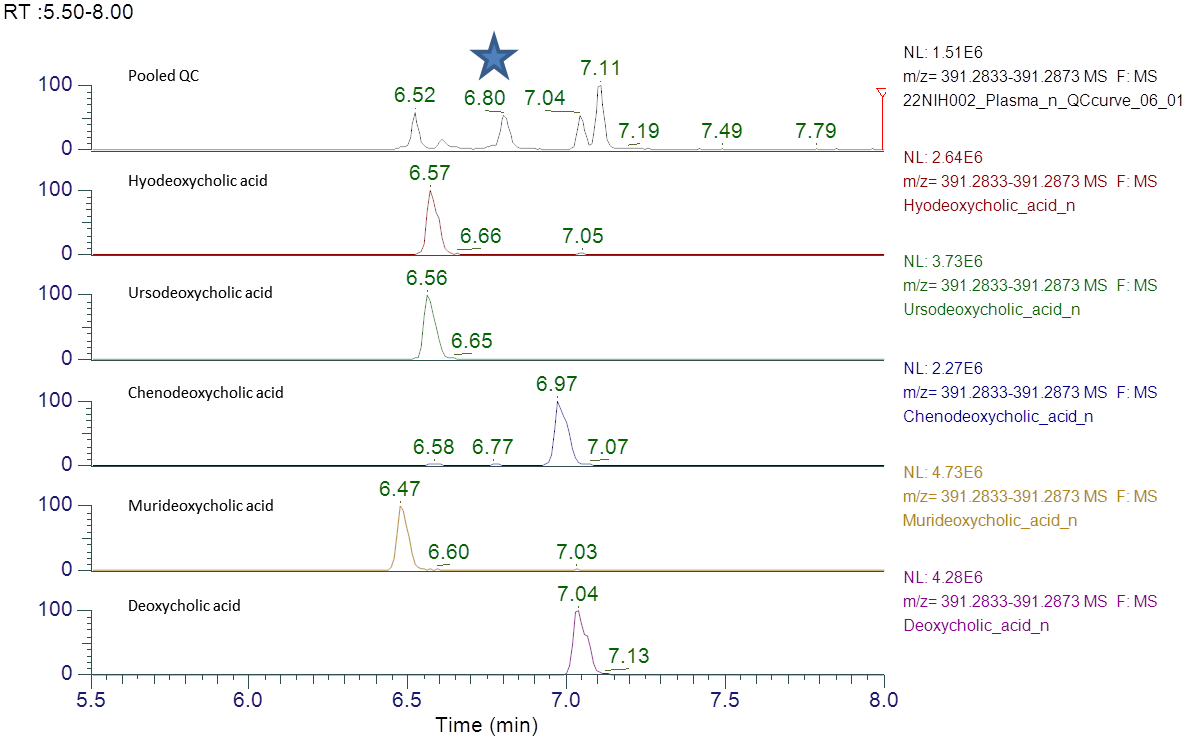
**

Chromatographic retention time of each in-house annotated bile acid feature (shown in pooled QC, top row) compared to chromatographic retention time of possible mass matches in in-house reference standard library. Annotations were assigned as the analyte with the closest retention time match, and the significant analyte peak annotated with a star.

## Supplementary Figure 5. Cholic secondary bile acid organic structures.


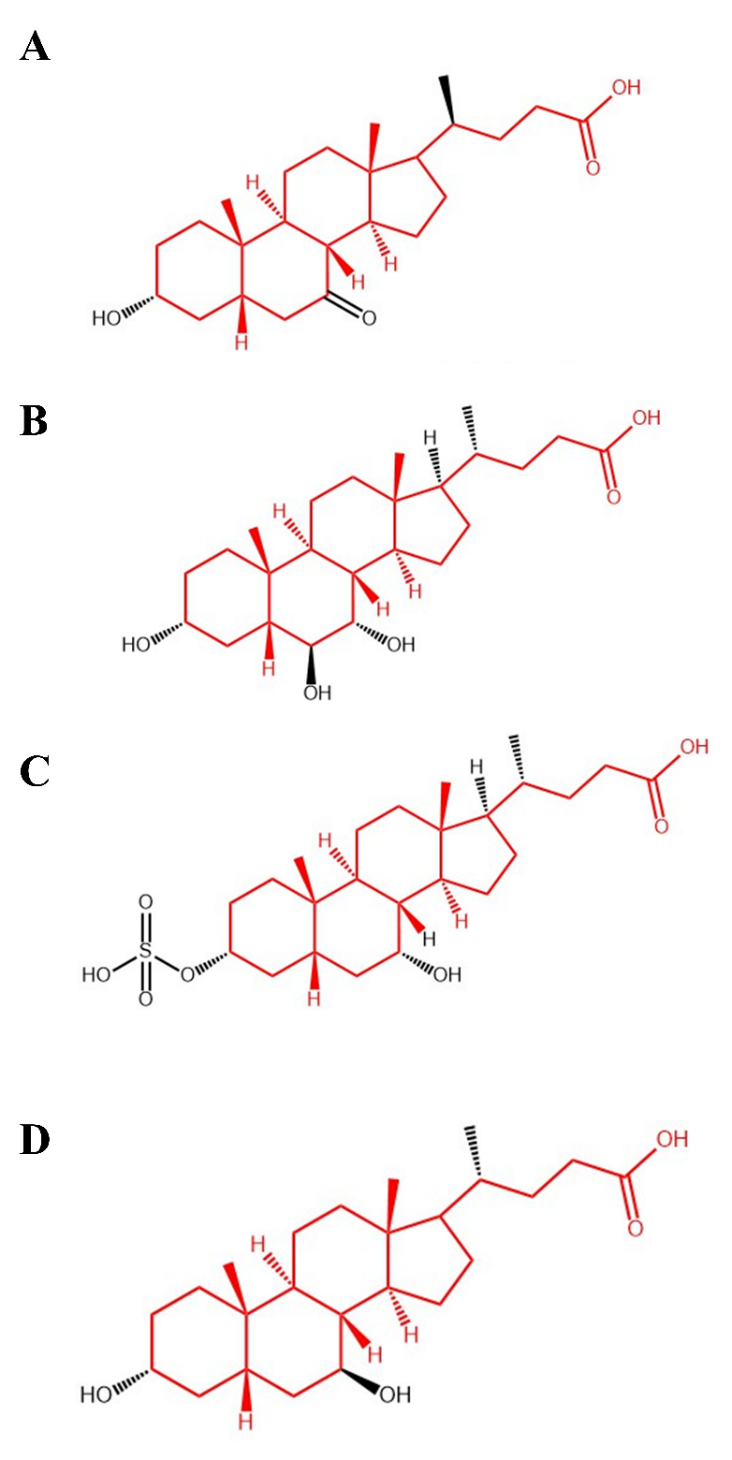


The similarities among the structures are shown in red and the differences between the structures are shown in black. **(A)** Nutriacholic Acid (Feature 4925|389.2696|6.83). **(B)** Alpha-Muricholic Acid (Feature 6219|407.2801|6.06). **(C)** Sulfochenodeoxycholic Acid (Feature 1400|471.242|6.11).

## Supplementary Figure 6. Microbiome signatures before (Pre-Met) and after metformin (Post-Met).


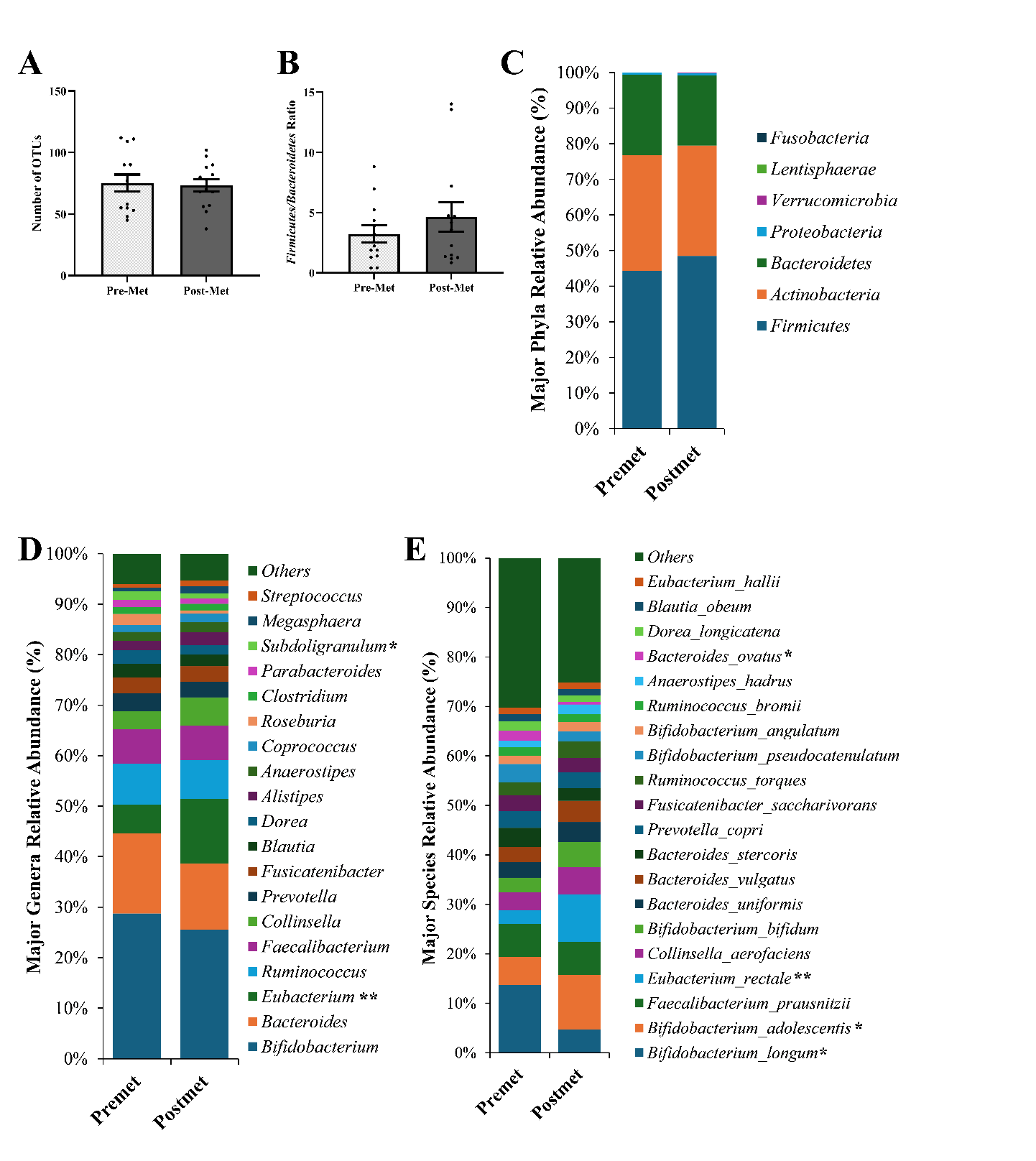


**(A)** Bar charts showing the number of Operational Taxonomic Units (OTUs) determined by 16S rRNA gene sequencing did not differ between Pre-Met versus Post-Met. Data shown are mean and standard error of mean. **(B)** Bar charts showing the change in the *Firmicutes/Bacteroidetes* ratio did not differ between Pre-Met versus Post-Met. Data shown are mean and standard error of mean. **(C-E)** Bar charts show the relative abundance of major phyla did not differ, but the genera and species differed between Pre-Met versus Post-Met. The number of OTUs, *Firmicutes/Bacteroides* ratio, and bacterial proportions were compared using the Kruskal-Wallis test followed by the Mann-Whitney multiple pairwise comparison test. Statistical significance was determined as **P<0.05*; ***P<0.01*. Pre-Met (n=13) and Post-Met (n=14).

## Supplementary Figure 7. Microbiome signatures before (Pre-Met+Lira) and after metformin and liraglutide (Post-Met+Lira).


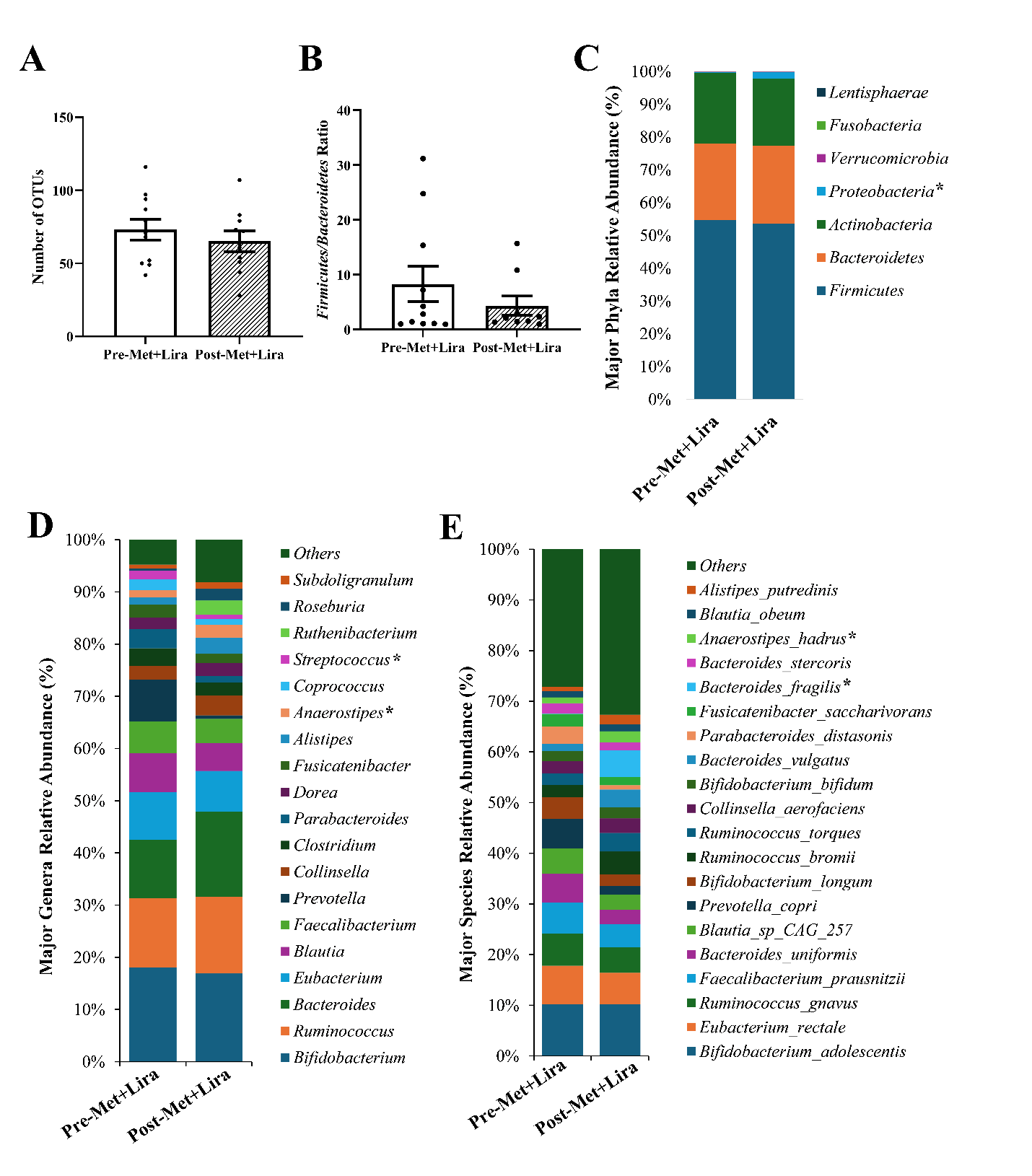


**(A)** Bar charts showing the number of Operational Taxonomic Units (OTUs) determined by 16S rRNA gene sequencing did not differ between Pre-Met+Lira versus Post-Met+Lira. Data shown are mean and standard error of mean. **(B)** Bar charts showing the change in the *Firmicutes/Bacteroidetes* ratio did not differ between Pre-Met+Lira versus Post-Met+Lira. Data shown are mean and standard error of mean. **(C-E)** Bar charts show the relative abundance of major phyla, genera, and species differed between Pre-Met+Lira versus Post-Met+Lira. The number of OTUs, *Firmicutes/Bacteroides* ratio, and bacterial proportions were compared using the Kruskal-Wallis test followed by the Mann-Whitney multiple pairwise comparison test. Statistical significance was determined as **P<0.05*; ***P<0.01*. Pre-Met+Lira (n=11) and Post-Met+Lira (n=9).

# References

1. Sumner LW, Amberg A, Barrett D, Beale MH, Beger R, Daykin CA, Fan TW, Fiehn O, Goodacre R, Griffin JL *et al*: **Proposed minimum reporting standards for chemical analysis Chemical Analysis Working Group (CAWG) Metabolomics Standards Initiative (MSI)**. *Metabolomics* 2007, **3**(3):211-221.
